# Research on the intelligent detection model of plant diseases based on MamSwinNet

**DOI:** 10.3389/fpls.2025.1676148

**Published:** 2025-10-31

**Authors:** Ao Zhang, Wei Liu

**Affiliations:** School of Information Science and Engineering, Shenyang Ligong University, Shenyang, China

**Keywords:** plant disease detection, deep learning, multi-scale feature extraction, image processing, lightweight mode

## Abstract

Plant diseases pose a severe threat to global agricultural production, significantly challenging crop yield, quality, and food security. Therefore, accurate and efficient disease detection is crucial. Current detection methods have clear limitations: CNN-based methods struggle to model long-range dependencies effectively and have weak generalization abilities. Transformer-based methods, while adept at long-range feature modeling, face issues with large parameter sizes and inefficient calculations due to the quadratic complexity of the self-attention mechanism in relation to image size. To address these challenges, this paper proposes the MamSwinNet model. Its core innovation lies in: using the Efficient Token Refinement module with an overlapping space reduction method, relying on depthwise separable convolutions designed with “stride + 3” convolution kernels to expand the image block overlap area and fully preserve boundary spatial structure. This generates high-quality tokens and converts them into a fixed number of latent tokens, reducing computational complexity while maximizing the retention of key features. It integrates the Spatial Global Selective Perception (SGSP) module and the Channel Coordinate Global Optimal Scanning (CCGOS) module. The SGSP module uses a dual-branch structure (the spatial modeling branch introduces 2D-SSM to scan four directions for capturing long-range dependencies, and the residual compensation branch supplements features to prevent loss; the two branches are combined using Hadamard product to enhance spatial detail modeling). The CCGOS module combines channel and spatial attention by embedding positional information through global average pooling in the height and width dimensions, using the Mamba block for channel-selective scanning and generating an attention map, enabling precise association of key channel features like color with spatial distribution. Experimental results show that the model achieves F1 scores of 79.47%, 99.52%, and 99.38% on the PlantDoc, PlantVillage, and Cotton datasets, respectively. The model has only 12.97M parameters (52.9% less than the Swin-T model) and a computational cost as low as 2.71GMac, significantly improving computational efficiency. This study provides an efficient and reliable intelligent solution for large-scale crop disease detection.

## 1 Introduction

Plant diseases represent a major challenge to modern agriculture, posing severe threats to global food production and security. With the continuous development of agricultural technologies, traditional manual detection methods, characterized by strong subjectivity and high labor costs, have proven insufficient for the needs of large-scale production. Consequently, crop disease detection is rapidly shifting toward automation and intelligence, driven in particular by breakthroughs in image processing and machine learning.

In early studies, Panchal et al. (2019) proposed a machine-learning-based approach for plant disease detection. Their method employed K-means clustering for image segmentation, gray-level co-occurrence matrices (GLCM) for feature extraction, and classification through HSV classifiers and random forests. The results achieved an accuracy of 98%, demonstrating the potential of machine learning to enhance classification precision. Similarly, Phadikar (2012) focused on morphological features of leaves, analyzing the tonal distribution of diseased foliage and using Bayesian classifiers and SVMs to identify brown spot and rice blast with accuracies of 79.5% and 68.1%, respectively, thereby validating the effectiveness of morphology-based analysis. Duhan and colleagues, (2024) introduced an integrated recognition strategy combining multiple machine learning techniques, including preprocessing, segmentation, and feature extraction. Their work showed that enhancing dataset diversity and image quality significantly improves detection accuracy. Kusumo et al. (2018) applied SVM and K-nearest neighbor (KNN) with image segmentation and GLCM-based features to classify maize diseases, achieving an accuracy of 92.7%, further underscoring the importance of visual features in detection.

As technology advanced, research gradually shifted toward more sophisticated deep learning models. Ashurov et al. (2025) incorporated squeeze-and-excitation (SE) modules and optimized residual connections into CNNs, raising accuracy to 98% with an F1-score of 98.2%, effectively balancing performance and efficiency. Ouamane et al. (2024) employed tensor subspace learning with HOSVD-MDA to design CNNs for tomato disease detection, achieving an accuracy of 92.6% and illustrating the benefit of dimensionality reduction in handling complex image classification tasks. Rakib et al. (2024) proposed a lightweight Q-CNN model tailored for IoT devices, enabling the recognition of nine diseases at 98% accuracy even in resource-constrained environments, thus providing a practical solution for intelligent agricultural devices. Bhargava et al. (2024) developed a CNN-based system with data augmentation that performed robustly across multiple plant species and disease types.

In recent years, the success of Transformer architectures has inspired new directions for plant disease detection. Visual Transformer (ViT) models have proven effective in image classification tasks, opening new opportunities for this domain. Zhang et al. (2021) introduced a Swin-Transformer-based model for rice disease recognition, leveraging shifted windows and hierarchical design to achieve 93.4% accuracy, which represented a 4.1% improvement over traditional models. Ali et al. (2025) utilized ViT’s self-attention to capture subtle lesion features, significantly improving recognition accuracy. Baek (2025) proposed the Multi-ViT model, integrating multiple ViTs to enhance classification on apple, grape, and tomato datasets, achieving F1-scores exceeding 90%.

Overall, existing methods for plant disease detection fall into three main categories, each with unique strengths but also evident limitations. Traditional machine learning approaches are computationally efficient and interpretable but heavily rely on handcrafted features, limiting generalization to diverse disease patterns and large-scale applications. CNNs can automatically extract features and support lightweight deployment, yet their limited receptive field constrains their ability to capture long-range dependencies, reducing robustness in complex backgrounds while balancing accuracy and efficiency remains difficult. ViTs, with their self-attention mechanism, excel in long-range modeling and are particularly sensitive to subtle lesions; however, their quadratic computational complexity with respect to image size imposes excessive overhead, restricting deployment on resource-limited platforms such as agricultural IoT devices. Thus, four major gaps remain: (1) traditional machine learning lacks adaptive generalization for complex lesion patterns; (2) CNNs struggle between local feature extraction and long-range dependency modeling; (3) Transformers face trade-offs between global modeling power, efficiency, and boundary preservation; (4) current methods are often limited to single-dimensional features, failing to achieve channel–spatial–cross-dimensional synergy.

To address these challenges, this study proposes MamSwinNet, a novel model built upon three innovative modules. (1) The Efficient Token Refinement module employs stride+3 depthwise separable convolutions to enlarge patch overlap, preserving lesion boundary information while refining token selection to reduce redundancy. This lowers parameter count to 12.97M (a 52.9% reduction compared with Swin-T) and computational cost to 2.71 GMac. (2) The SGSP module integrates four-directional 2D-SSM scanning with residual branches to strengthen spatial dependency modeling and enhance complex lesion recognition. (3) The CCGOS module fuses channel–spatial attention using global average pooling and Mamba blocks, thereby improving robustness. Experimental results demonstrate that MamSwinNet achieves F1-scores of 79.47%, 99.52%, and 99.38% on the PlantDoc, PlantVillage, and Cotton datasets, respectively, effectively overcoming the limitations of existing methods and achieving a balance between detection accuracy, computational efficiency, and adaptability to complex scenarios.

## 2 MamSwinNet model

The Swin Transformer is a hierarchical vision Transformer architecture whose central innovations are window-based self-attention (W-MSA) and the shifted window scheme (SW-MSA). In W-MSA, the input image is evenly partitioned into non-overlapping local windows, and self-attention is computed within each window rather than globally across the entire image. This design reduces the quadratic complexity of traditional ViTs to a linear relationship with the number of windows, eliminating redundant computations while accurately capturing fine-grained local details. The SW-MSA mechanism addresses the “information isolation” caused by fixed local windows. By shifting the window partition in successive Transformer layers (e.g., half-window displacement), features originally separated into different windows are merged into the same window, enabling cross-window information flow and feature interaction. The combination of W-MSA and SW-MSA ensures that Swin Transformer achieves an effective balance between computational efficiency and global modeling capability. Moreover, with its hierarchical representation and Patch Merging, Swin progressively constructs multi-scale feature pyramids well-suited to dense prediction tasks.

However, in the specific context of plant disease detection, Swin Transformer still faces three major limitations. First, its fixed, non-overlapping patch partitioning may fragment lesion boundary structures, while redundant background tokens make it difficult to jointly optimize computational efficiency and lesion feature precision. Second, the locality of window-based attention restricts its ability to model long-range spatial dependencies, such as the spread of lesions across regions. Third, it lacks mechanisms to jointly correlate color features of lesions with their spatial positional attributes, reducing robustness under complex field conditions.

**Figure 1:**
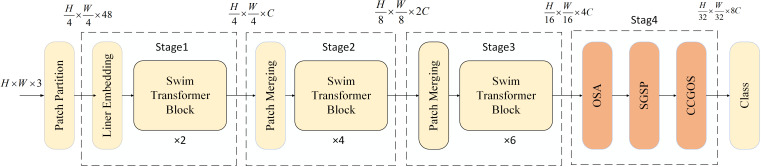
The four-stage feature extraction framework of the Swin-Mamba model.

To overcome these challenges, this study introduces MamSwinNet (**Figure 1**), a model built upon the principle of “hierarchical feature construction with efficient enhancement.” MamSwinNet retains the layered Swin backbone while incorporating three novel modules within a four-stage framework. The first three stages perform patch partitioning, linear embedding, multiple Swin Transformer blocks, and Patch Merging to gradually extract multi-scale hierarchical features, progressing from local textures to global structural representations: Stage 1 applies two blocks to the initial tokens, Stage 2 downsamples and processes with four blocks, and Stage 3 stacks six blocks while increasing dimensionality, yielding a feature pyramid that encodes both scale diversity and hierarchical depth. The fourth stage integrates three key modules: Efficient Token Refinement for reducing redundancy while preserving lesion boundaries and balancing accuracy with efficiency; SGSP for capturing long-range spatial dependencies, such as cross-region lesion spread, through directional scanning with residual fusion; and CCGOS for channel–spatial coordination by integrating lesion color cues with positional information. Together, these modules enable precise extraction and efficient fusion of disease-related features, enhancing robustness and adaptability for complex plant disease detection scenarios.

### 2.1 Efficient token refinement module

In the field of plant disease detection, accurately identifying disease features is crucial for ensuring agricultural production safety. However, the complexity and diversity of disease features (such as irregular shapes of lesions and subtle differences in color) pose a significant challenge for feature extraction. The current mainstream Transformer models adopt a fixed-size image partitioning strategy, which, although capable of capturing global features, fails to retain key boundary information of the image when processing high-resolution disease images. This results in inefficient extraction of high-quality tokens and leads to a large amount of redundant computation. Not only does this increase computational complexity and memory consumption, but it also makes real-time detection more difficult.

**Figure 2:**
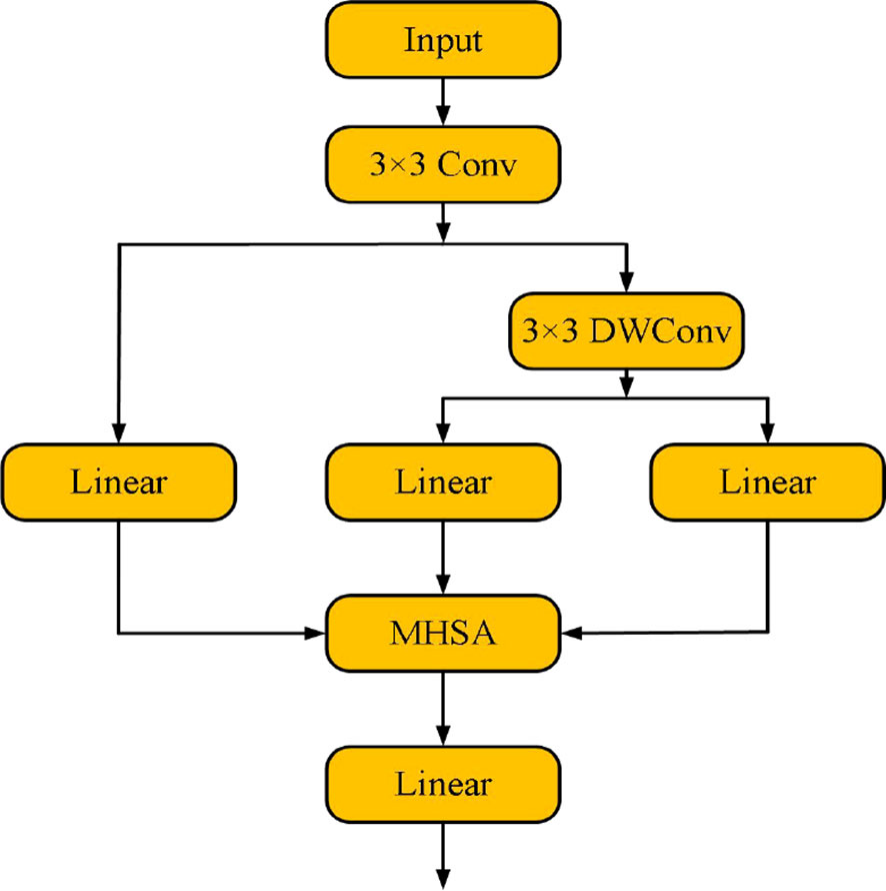
Efficient token refinement module structure.

To address this issue, this paper proposes an innovative Efficient Token Refinement module (as shown in **Figure 2**). First, an overlapping spatial reduction method is introduced, relying on deep separable convolutions. The convolution kernel size is designed as “stride + 3”, and by expanding the overlapping region of image blocks, this method effectively represents the boundary spatial structure. This design allows for more complete retention of boundary information during spatial reduction, thereby improving the quality of token features and the integrity of their spatial structure. Secondly, high-quality tokens are converted into a fixed number of latent tokens. This process reduces computational complexity while maximizing the retention of key features, providing strong support for the efficient processing of subsequent modules.

First, the Efficient Token Refinement module performs preliminary processing on input features through a 3×3 convolution, aiming to extract the feature map ${\text{F}}_{\text{init}}$ with basic spatial information and adjust the number of channels to match the subsequent embedding dimension. The initial convolution operation is expressed by the following formula:


$${\text{F}}_{\text{init}}\;=\text{ Conv}3\times 3\;{\text{(X), F}}_{\text{init}}\;\in {\mathbb{R}}^{\text{B}\times \text{C}\times \text{H}\times \text{W}}$$

Among them, B、C、H、and W represent the batch size, number of channels, height, and width, respectively.

On this basis, ${\text{F}}_{\text{init}}$ first adopts a depthwise convolution with a kernel size of “stride + 3” to carry out spatial dimension reduction on the feature map, obtaining the feature representation $\text{Y}\in {\mathbb{R}}^{\text{B}\times \text{C}\times {\text{H}}^{\prime }\times {\text{W}}^{\prime }}$ after spatial reduction.

Subsequently, the local refinement module constructed by 3×3 depthwise convolution is utilized to process Y Through linear transformation and splitting operations, Y is divided into $\text{K}\in {\mathbb{R}}^{\text{B}\times \text{C}\times {\text{H}}^{\prime }\times {\text{W}}^{\prime }}$ and $\text{V}\in {\mathbb{R}}^{\text{B}\times \text{C}\times {\text{H}}^{\prime }\times {\text{W}}^{\prime }}$ , which is formulated as:


K,V=Split(Linear(Y+LR(Y)))

For the query vector $\text{Q}\in {\mathbb{R}}^{\text{B}\times \text{C}\times \text{H}\times \text{W}}$ , it is generated by performing a linear transformation on the original input feature x, and its mathematical expression is:

T=Linear(x)

Based on (Q,K,V) , the softmax attention is calculated. The output of the attention mechanism $\text{Z}\in {\mathbb{R}}^{\text{C}\times \text{H}\times \text{W}}$ is realized through the following formula:


$$\text{Z}=\text{Softmax}(\frac{{\text{QK}}^{\text{T}}}{\sqrt{\text{d}}}+\text{B})\text{V}$$

Among them,  B is the relative position bias, which is used to encode the spatial correlation of the spatial attention map.

Finally, the module maps the enhanced features into a fixed number of latent tokens through a linear transformation layer. The specific process is as follows:


$$\text{T}=\text{Linear}({\text{F}}_{\text{att}}),\text{T}\in {\mathbb{R}}^{\text{B}\times \text{T}\times \text{E}}$$

Among them, T is the number of tokens, and  E is the embedding dimension of each token. Through this process, the module significantly reduces computational complexity, while retaining key multi-scale and global features, providing a more efficient input representation for subsequent modules.

### 2.2 SGSP Module

**Figure 3:**
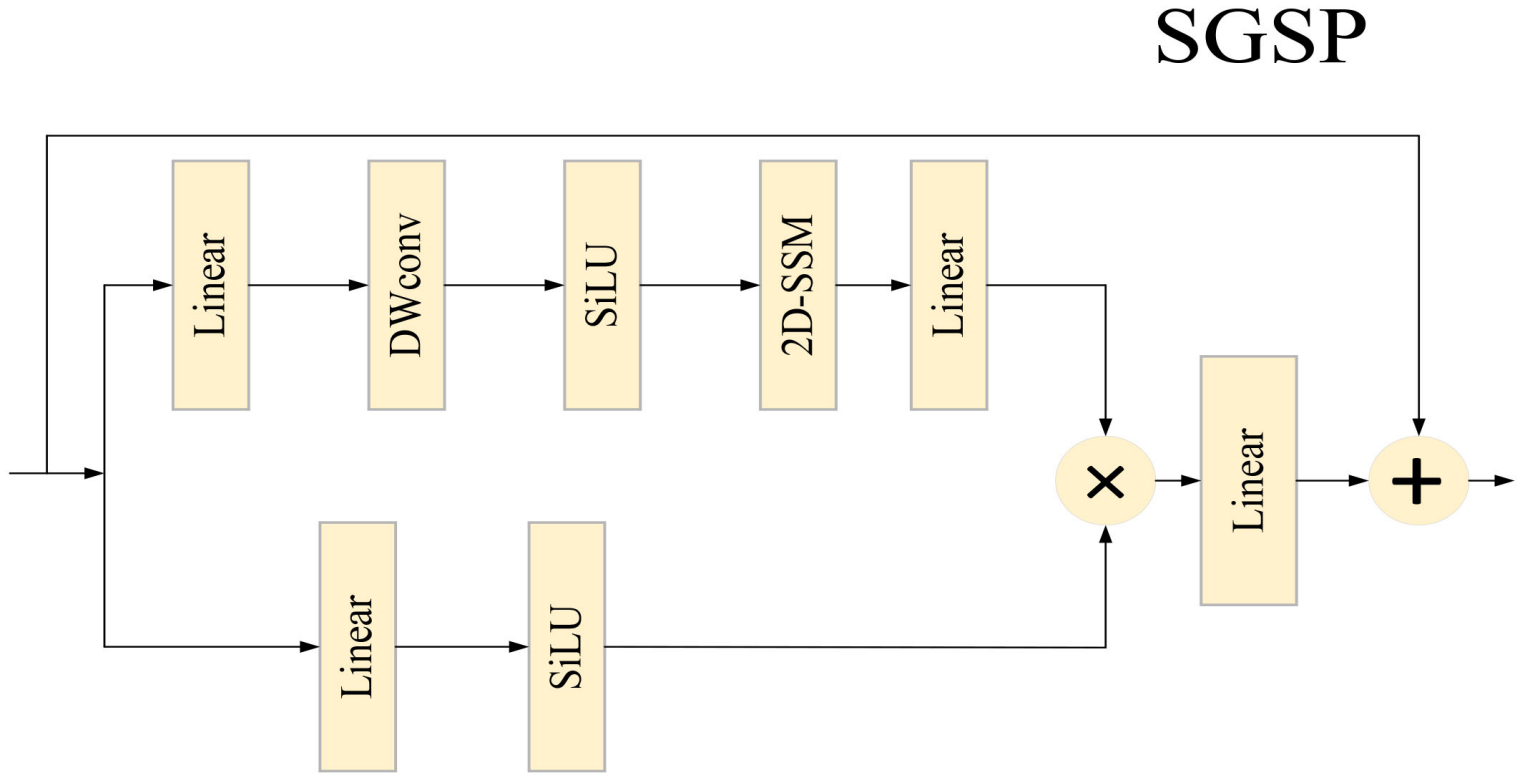
Schematic diagram of the SGSP module.

At the pixel - spatial dimension, to balance computational efficiency, Transformer - based plant disease and pest detection methods often adopt the shifted window attention mechanism. However, this mechanism has limited capability in modeling the global horizontal dimension of images, and plant disease and pest images generally suffer from the problem of easily lost global information. Inspired by the successful experience of Swin Transformer in achieving long - range modeling with linear complexity, we introduce the 2D Selective Spatial Scanning Module (2D - SSM) into the plant disease and pest detection task. As shown in **Figure 3**, the input feature $\text{X}\in {\text{R}}^{\text{C×H×W}}$ is processed through two parallel branches:

Branch 1 (Spatial Modeling Branch): First, channels are expanded via a linear layer, followed by depthwise convolution (DWConv), SiLU activation, and finally connected to the 2D - SSM layer with Layer Normalization (LN). The formula is:


$${\text{X}}_{1}=\text{LN}(2\text{D}-\text{SSM}(\text{SiLU}(\text{DWConv}(\text{Linear}(\text{X}))))$$

Among them, the 2D - SSM module first performs omnidirectional scanning on 2D image features along four directions: top - left to bottom - right, bottom - right to top - left, top - right to bottom - left, and bottom - left to top - right. After integrating the feature information captured by multi - direction scanning, it flattens the information into a one - dimensional sequence. Subsequently, it models and captures the long - range feature dependencies in the one - dimensional sequence using discrete state - space equations, and strengthens the transmission and association of key features through dynamic weight allocation. Finally, through feature summation, merging, and dimension reshaping operations, the processed features are restored to a 2D structure. This mechanism can adaptively focus on and capture key pixel information in images, effectively enhancing the network’s ability to model subtle spatial features in plant disease and pest images, and providing richer spatial detail support for the accurate identification of subsequent disease features.

Branch 2 (Residual Compensation Branch): It only expands channels via a linear layer and then applies SiLU activation. The formula is:


$${\text{X}}_{2}=\text{SiLU}(\text{Linear}(\text{X})),$$

The features of the two branches are aggregated via the Hadamard product ( ⊙ ). Finally, they are projected back to the input dimension and undergo residual fusion. The formula is:


$${\text{X}}_{\text{out}}=\text{Linear}({\text{X}}_{1}⨀{\text{X}}_{2})+\text{X}$$

### 2.3 CCGOS Module

When computing channel attention in image classification tasks based on Mamba, conventional methods often rely on global max pooling or average pooling operations. However, such approaches tend to lose spatial location information associated with the target region. To address this issue, we extend Mamba by introducing a channel attention modeling mechanism, further enhancing the spatial attention mechanism into a channel-spatial attention module. By embedding precise spatial location information, the network can more effectively focus on the most task-relevant channel features, thereby improving its perceptual capability.

**Figure 4:**
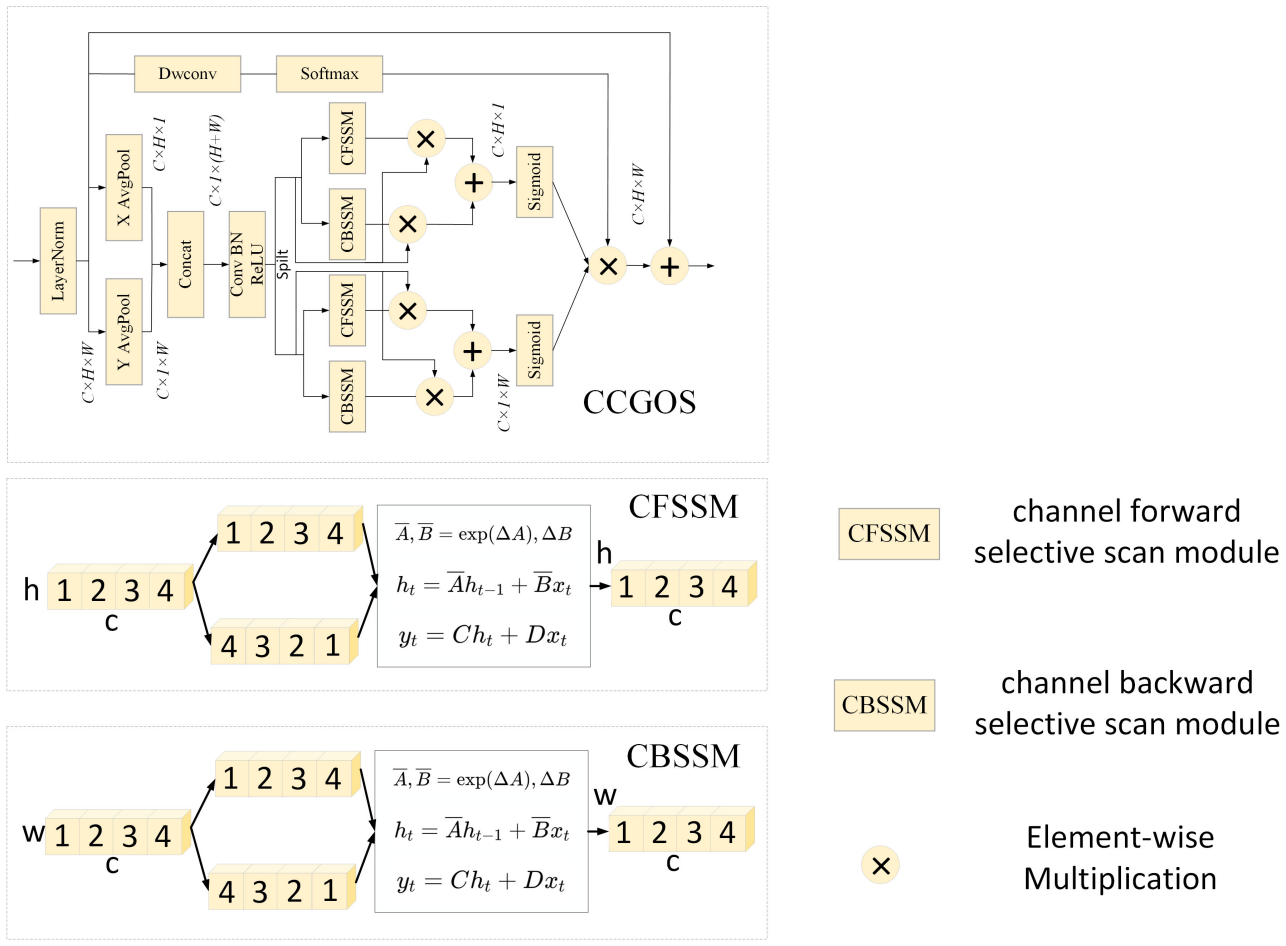
CCGOS Module Structure and Submodules (CFSSM/CBSSM) Working Principle Diagram.


As illustrated in **Figure 4**, given an input $\text{X}\in {\mathbb{R}}^{\text{C}\times \text{H}\times \text{W}}$ , average pooling is first applied along the height and width dimensions:

$${\text{X}}_{\text{h}}\in {\mathbb{R}}^{\text{C}\times 1\times \text{W}}={\text{AvgPool}}_{\text{h}}(\text{X})$$

$${\text{X}}_{\text{w}}\in {\mathbb{R}}^{\text{C}\times \text{H}\times 1}={\text{AvgPool}}_{\text{w}}(\text{X})$$

The results are then concatenated and processed sequentially through a Conv-BN-ReLU operation, after which the feature map is split back into two components:


$${\text{X}}_{\text{hw}}\in {\mathbb{R}}^{\text{C}\times 1\times (\text{H}+\text{W})}=\text{ReLU}(\text{BN}(\text{Conv}(\text{Concat}({\text{X}}_{\text{h}},{\text{X}}_{\text{w}}))))$$

$${\text{X}}_{\text{h}}\in {\mathbb{R}}^{\text{C}\times 1\times \text{W}},{\text{X}}_{\text{w}}\in {\mathbb{R}}^{\text{C}\times \text{H}\times 1}=\text{Split}({\text{X}}_{\text{hw}})$$

Next, ${\text{W}}_{\text{h}}$ and ${\text{W}}_{\text{w}}$ are fed into the channel-selective scanning module, which consists of two parts: the Channel Forward Selective Scanning Module (CFSSM) and the Channel Backward Selective Scanning Module (CBSSM). After passing through the Sigmoid activation, the height and width attention weights ${\text{W}}_{\text{h}}$ and ${\text{W}}_{\text{w}}$ are obtained:


$${\text{W}}_{\text{h}}=\text{Sigmoid}(\text{CFSSM}({\text{X}}_{\text{h}})⨀({\text{X}}_{\text{h}})+\text{CBSSM}({\text{X}}_{\text{h}})⨀({\text{X}}_{\text{h}}))$$

$${\text{W}}_{\text{w}}=\text{Sigmoid}(\text{CFSSM}({\text{X}}_{\text{w}})⨀({\text{X}}_{\text{w}})+\text{CBSSM}({\text{X}}_{\text{w}})⨀({\text{X}}_{\text{w}}))$$

Finally, the adjusted channel features with ${\text{W}}_{\text{h}}$ and ${\text{W}}_{\text{w}}$ are combined through a 1×1 convolution and a Softmax activation function to produce the output of the CCGOS module:


$$\text{Y}\in {\mathbb{R}}^{\text{C}\times \text{H}\times \text{W}}={\text{W}}_{\text{w}}⨀{\text{W}}_{\text{h}}①\text{Softmax}(\text{Conv}(\text{X}))+\text{X}$$

Through this design, the CCGOS module not only preserves global contextual information but also embeds spatial coordinate information during the modeling process, thereby addressing the limitations of traditional pooling-based attention mechanisms in representing spatial distributions. While maintaining computational efficiency, the module significantly enhances the model’s ability to perceive and classify complex disease features.

## 3 Experiments

### 3.1 Experimental datasets

This study selected three representative datasets—PlantDoc, PlantVillage, and Cotton Disease—to systematically validate the effectiveness and robustness of the proposed model. All datasets followed a uniform partitioning strategy, randomly divided into training, validation, and test sets in an 8∶1∶1 ratio. To mitigate the influence of randomness on the experimental outcomes, each experiment was independently repeated five times, and the median performance across repetitions was adopted as the final evaluation metric, thereby ensuring the stability and scientific rigor of the results.

1. PlantDoc Dataset

**Figure 5:**
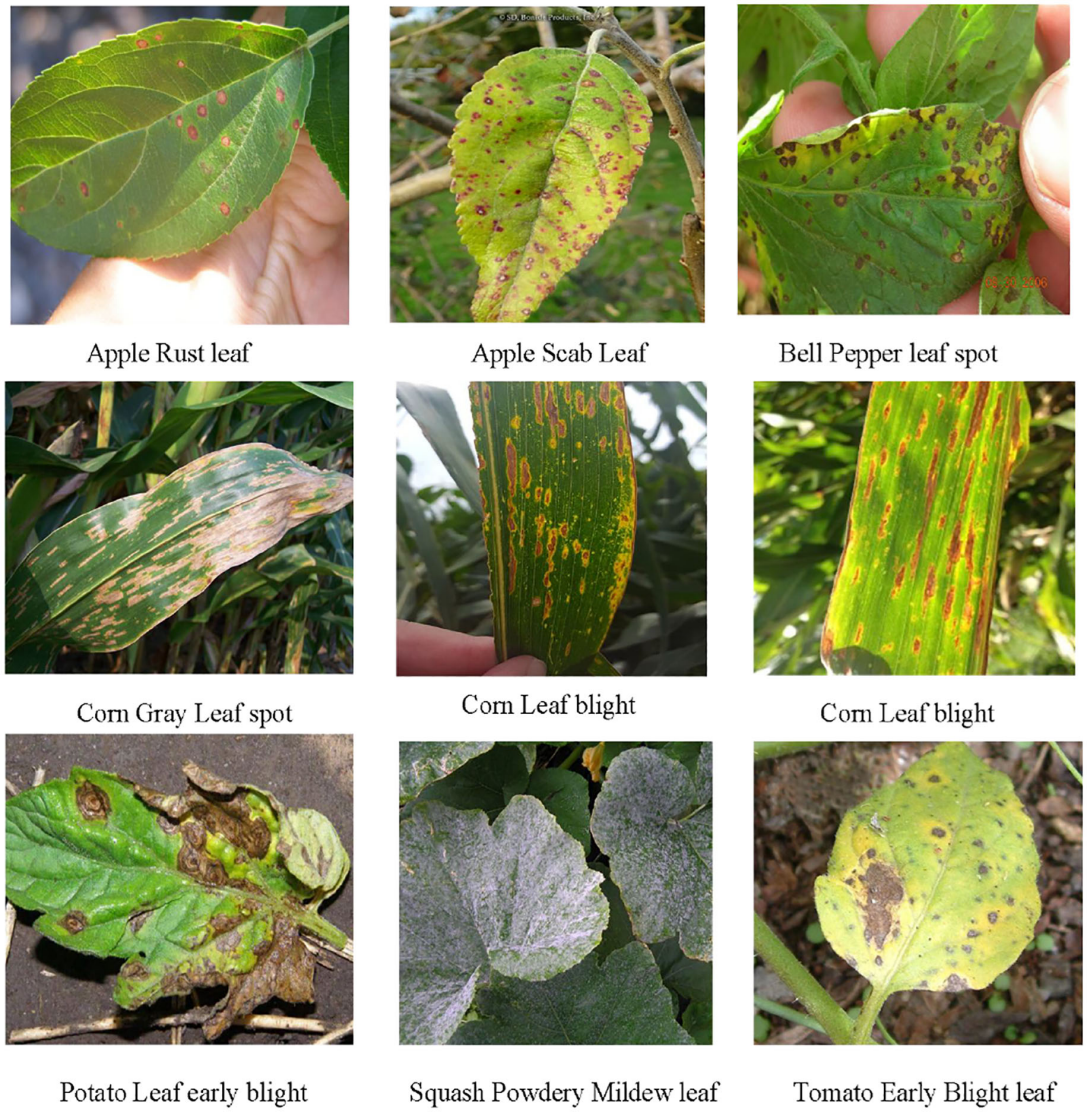
Sample images from the PlantDoc dataset.

The PlantDoc dataset is a high-quality open-source resource specifically developed for plant disease detection tasks, providing real-world samples collected under natural field conditions. It encompasses 13 major crops (such as maize, tomato, and wheat) and 27 representative plant diseases (including rust, powdery mildew, and leaf spot), all of which are precisely annotated by experts. Compared with laboratory-collected data, PlantDoc images more faithfully capture the complexity and diversity of plant disease manifestations in natural environments, including variations in lighting, background interference, and lesion morphology. This realism considerably increases the difficulty of the recognition task while enhancing its practical relevance. Owing to these characteristics, PlantDoc has become a key benchmark dataset for evaluating the robustness and applicability of plant disease detection models. **Figure 5** illustrates several representative samples from the dataset. The dataset is publicly accessible via the following link: https://github.com/pratikkayal/PlantDoc-Dataset

2. PlantVillage Dataset

**Figure 6:**
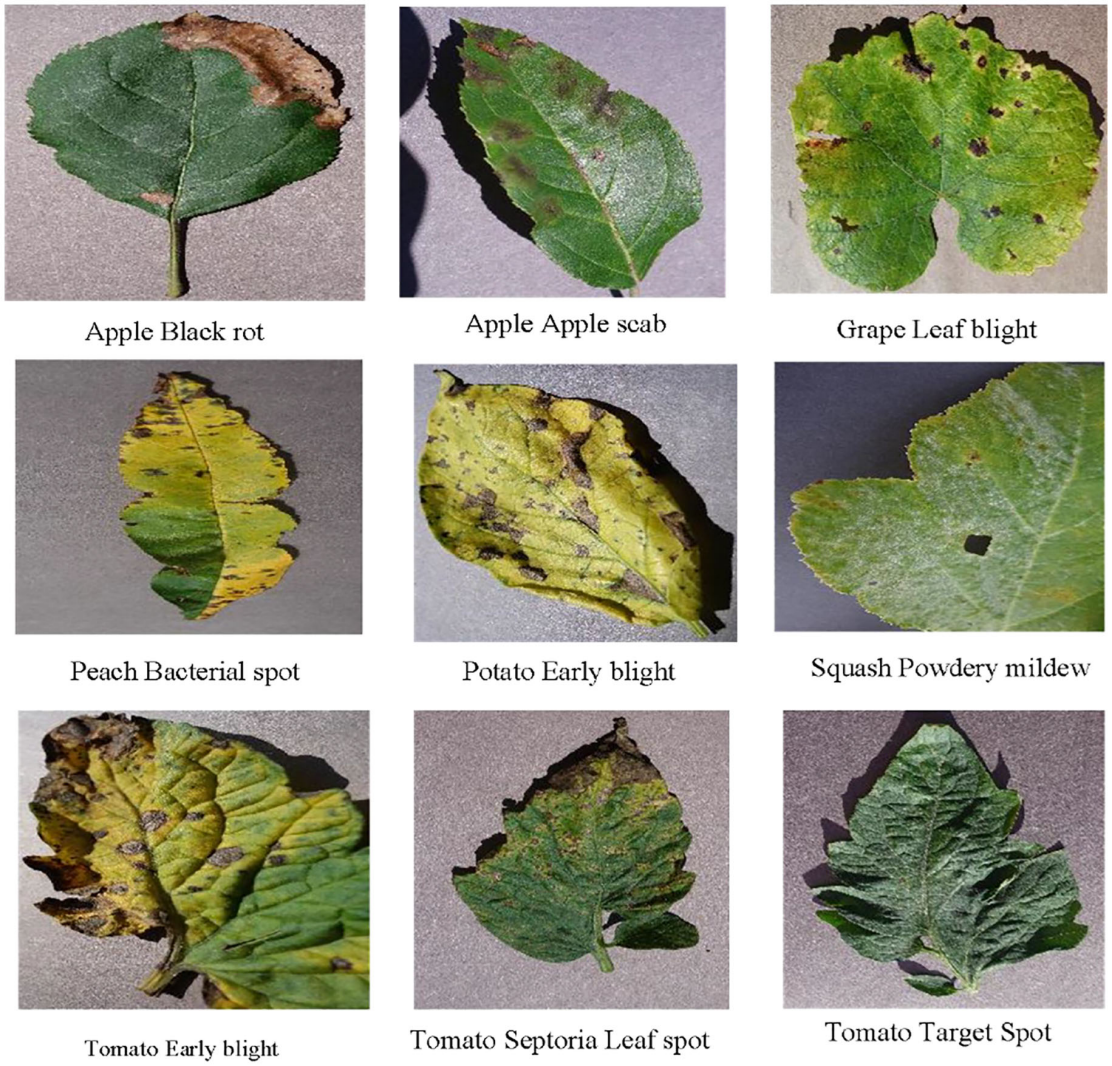
Sample images from the PlantVillage dataset.

The PlantVillage dataset is one of the most widely used open-source datasets for leaf disease recognition, designed and released by the Spandan Mohanty team. It includes 14 major crops (such as tomato, apple, and maize) and 38 disease categories. All samples were collected under controlled laboratory conditions, characterized by clean backgrounds, uniform illumination, and clearly visible lesions. This standardized acquisition method ensures high data quality, facilitating the training and validation of deep learning models. Thanks to its advantages of environmental controllability and distinct disease features, PlantVillage is frequently employed for model pre-training and performance benchmarking, and has become an important reference dataset in plant disease detection research. **Figure 6** presents several representative samples from this dataset. PlantVillage can be accessed via the following link: https://www.kaggle.com/datasets/abdallahalidev/plantvillage-dataset

3. Cotton Disease Dataset

**Figure 7:**
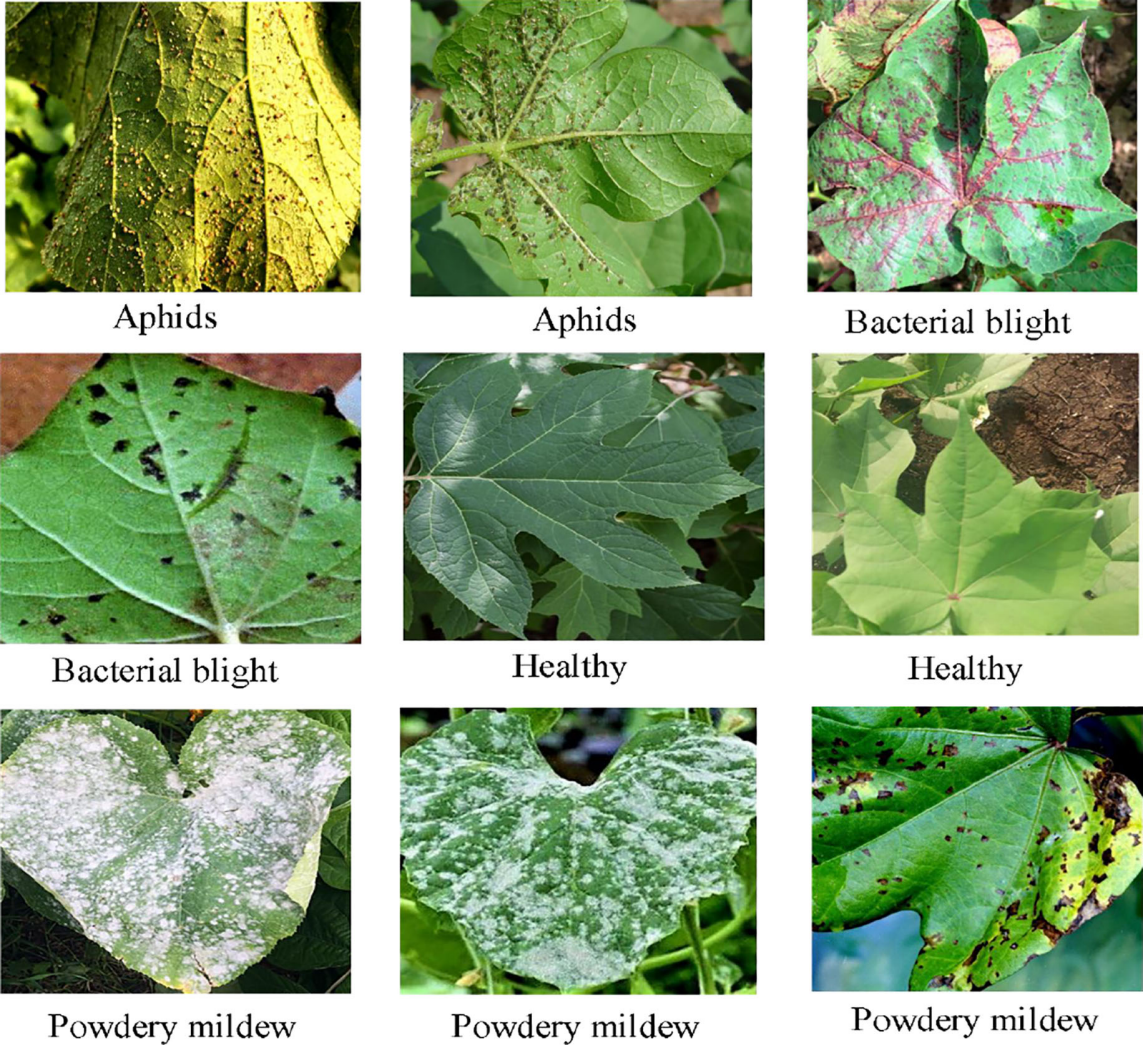
Sample images from the cotton disease dataset.

The Cotton Disease dataset is dedicated to research on cotton plant diseases, with a particular focus on the automatic recognition of cotton leaf diseases. It includes five representative disease categories—aphid infestation, bollworm damage, bacterial blight, powdery mildew, and target spot—while also incorporating healthy leaf images as control samples. The dataset’s class design not only covers the major and prevalent diseases in cotton production but also provides a reliable benchmark for evaluating models in multi-class disease classification tasks. **Figure 7** presents several representative image samples from this dataset. The dataset is publicly available at the following link: https://www.kaggle.com/datasets/dhamur/cotton-plant-disease.

### 3.2 Experimental environment and evaluation metrics

The experiments in this study were conducted under the following hardware and software settings to ensure efficient training and inference of the deep learning models. The hardware platform comprised an NVIDIA RTX 2080Ti GPU with 12 GB of memory and an Intel Xeon Platinum 8336C CPU. The software environment was configured with Python 3.8 and PyTorch 1.11.0, with model training implemented on CUDA 11.3.All experiments were standardized with an input image size of 224 × 224, a batch size of 50, and an initial learning rate of 5e-5. The AdamW optimizer was adopted for training. To comprehensively evaluate the performance of the proposed MamSwinNet model in plant disease recognition, several evaluation metrics were employed. These include Precision, Recall, and F1-Score to assess classification performance, as well as model complexity indicators—number of parameters and GMACs (Giga Multiply–Accumulate operations)—to measure computational efficiency. The definitions of these metrics are as follows:

1. Precision

Precision measures the proportion of samples predicted as diseased that are actually diseased. It is defined as:


$$\text{Precision}=\frac{\text{TP}}{\text{TP}+\text{FP}}$$

where TP (True Positive) represents the number of samples correctly predicted as diseased, and FP (False Positive) denotes the number of healthy samples incorrectly predicted as diseased. A higher precision indicates that the model makes fewer errors when classifying healthy samples as diseased, thereby reducing unnecessary pesticide application and resource waste.

2. Recall

Recall measures the proportion of truly diseased samples that are correctly identified by the model. It is defined as:


$$\text{Recall}=\frac{\text{TP}}{\text{TP}+\text{FN}}$$

where FN (False Negative) refers to the number of diseased samples incorrectly predicted as healthy. A higher recall reflects the model’s ability to capture more diseased samples, thereby reducing the risk of missed detections. This is especially important in detecting severe plant diseases, where higher recall helps in timely warning and prevention.

3. F1-Score

The F1-score integrates both Precision and Recall, balancing the trade-off between the two, and serves as a comprehensive indicator of model performance. It is defined as:


$$\text{F}1-\text{Score}=2\times \frac{\text{Precision}\times \text{Recall}}{\text{Precision}+\text{Recall}}$$

The F1-score ranges from 0 to 1, with higher values indicating better overall classification performance, especially in minimizing the trade-off between false alarms and missed detections. In this study, the F1-score is used as the key metric for evaluating the overall classification accuracy of plant disease detection.

4. Number of Parameters

This metric reflects the scale of trainable parameters within the model, measured in millions (M). A smaller parameter size indicates a lighter-weight model, which requires less storage and computational cost, making it more practical for deployment in resource-constrained agricultural scenarios.

5. GMACs

GMACs (Giga Multiply–Accumulate Operations) measure the computational complexity of the model during inference, with units representing billions of multiply–accumulate operations. A lower GMAC value implies faster inference speed and higher computational efficiency, which is essential for real-time plant disease detection and large-scale agricultural applications.

#### 3.2.1 Experimental results

In the validation experiments on the PlantDoc dataset, the MamSwinNet model demonstrated strong capability in classifying plant diseases under complex natural conditions, while also revealing certain limitations. As shown by the classification metrics (**Table 1**), the model performs well across most categories. Specifically, categories 15, 16, and 25 achieved Precision, Recall, and F1-Score values of 100%, indicating excellent discriminative power for disease types with distinct and representative features. Similarly, categories 9, 10, 13, 17, and 26 maintained overall metrics above 90%, further confirming the model’s stability and reliability in identifying mainstream disease types. However, several categories exhibited notably lower performance; for instance, categories 7 and 20 achieved F1-Scores of only 22.22% and 20.00%, respectively, while categories 3, 4, and 12 remained within the 50%–60% range. This disparity primarily arises from significant differences in the visual characteristics of various disease categories.

**Table 1:** Classification performance statistics of the PlantDoc dataset (by category).

Class	Precision (%)	Recall (%)	F1 Score (%)
0	90.00	90.00	90.00
1	77.78	77.78	77.78
2	100	80.00	88.89
3	66.67	50.00	57.14
4	62.50	55.56	58.83
5	75.00	81.82	78.26
6	87.50	70.00	77.78
7	20.00	25.00	22.22
8	69.23	75.00	72.00
9	100	90.00	94.74
10	100	88.89	94.12
11	53.84	87.50	66.66
12	75.00	37.50	50.00
13	87.50	100	93.33
14	60.00	75.00	66.67
15	100	100	100
16	100	100	100
17	90.00	100	94.74
18	50.00	100	66.67
19	100	62.50	76.92
20	100	11.11	20.00
21	77.78	70.00	73.69
22	85.71	60.00	70.59
23	75.00	100	85.71
24	60.00	100	75.00
25	100	100	100
26	100	87.50	93.33
ALL	81.50	77.54	79.47

**Figure 8:**
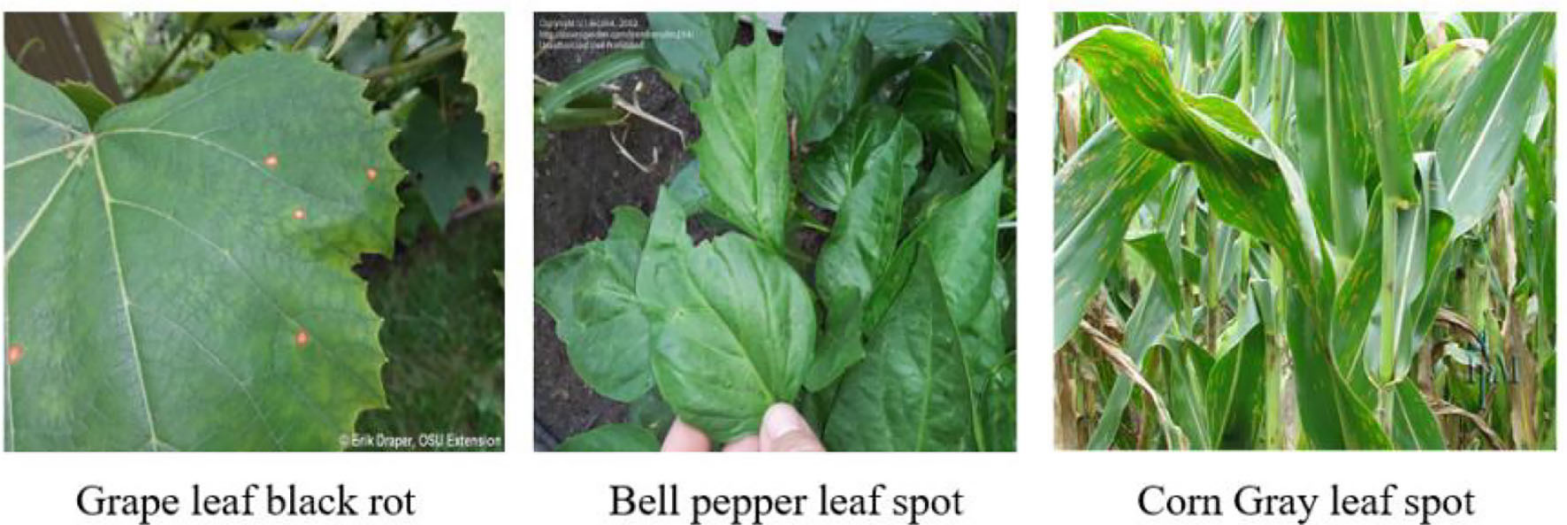
Representative samples of categories with insufficient recognition performance by the model.

**Figure 9:**
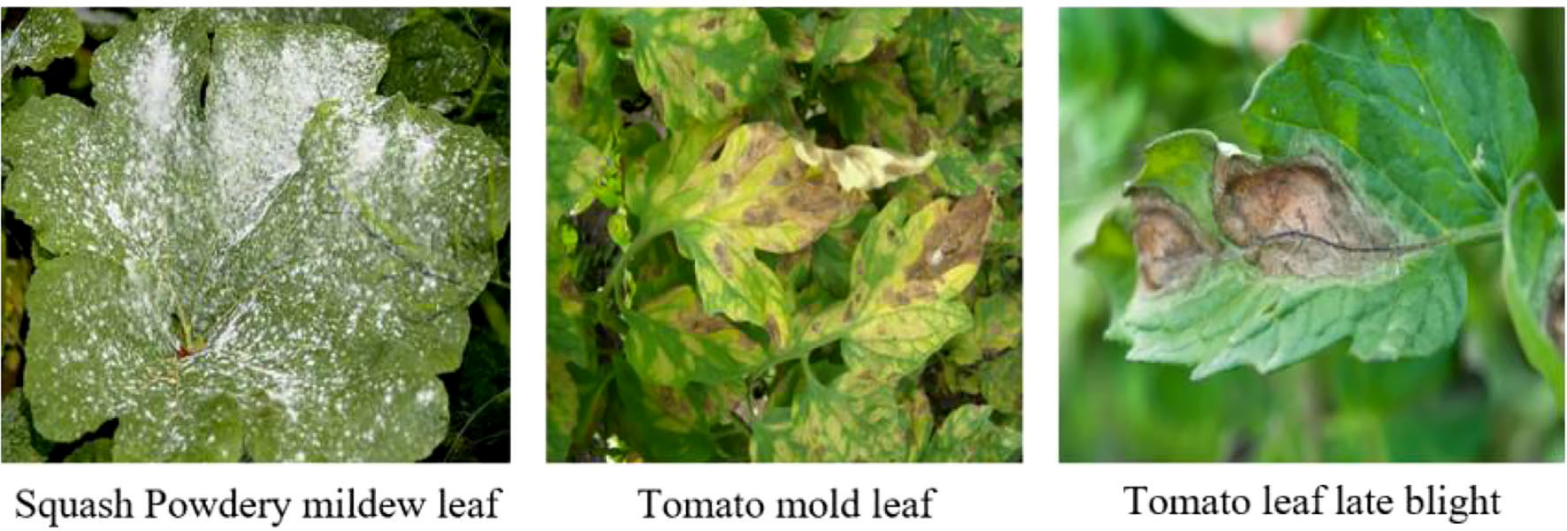
Representative samples of categories with superior recognition performance by the model.

As illustrated in **Figure 8**, for low-performing categories such as grape black rot, pepper leaf spot, and corn gray leaf spot, lesion colors are highly similar to the leaf background and exhibit blurred boundaries, making it difficult for the model to focus accurately on key lesion regions. In contrast, for high-performing categories such as pumpkin powdery mildew, tomato gray mold, and tomato late blight (**Figure 9**), the lesion boundaries are clear and the morphological features are distinctive, enabling the model to effectively capture discriminative regions and achieve precise classification. This phenomenon indicates that the visual separability between lesion and background is a critical factor affecting classification performance.

**Figure 10:**
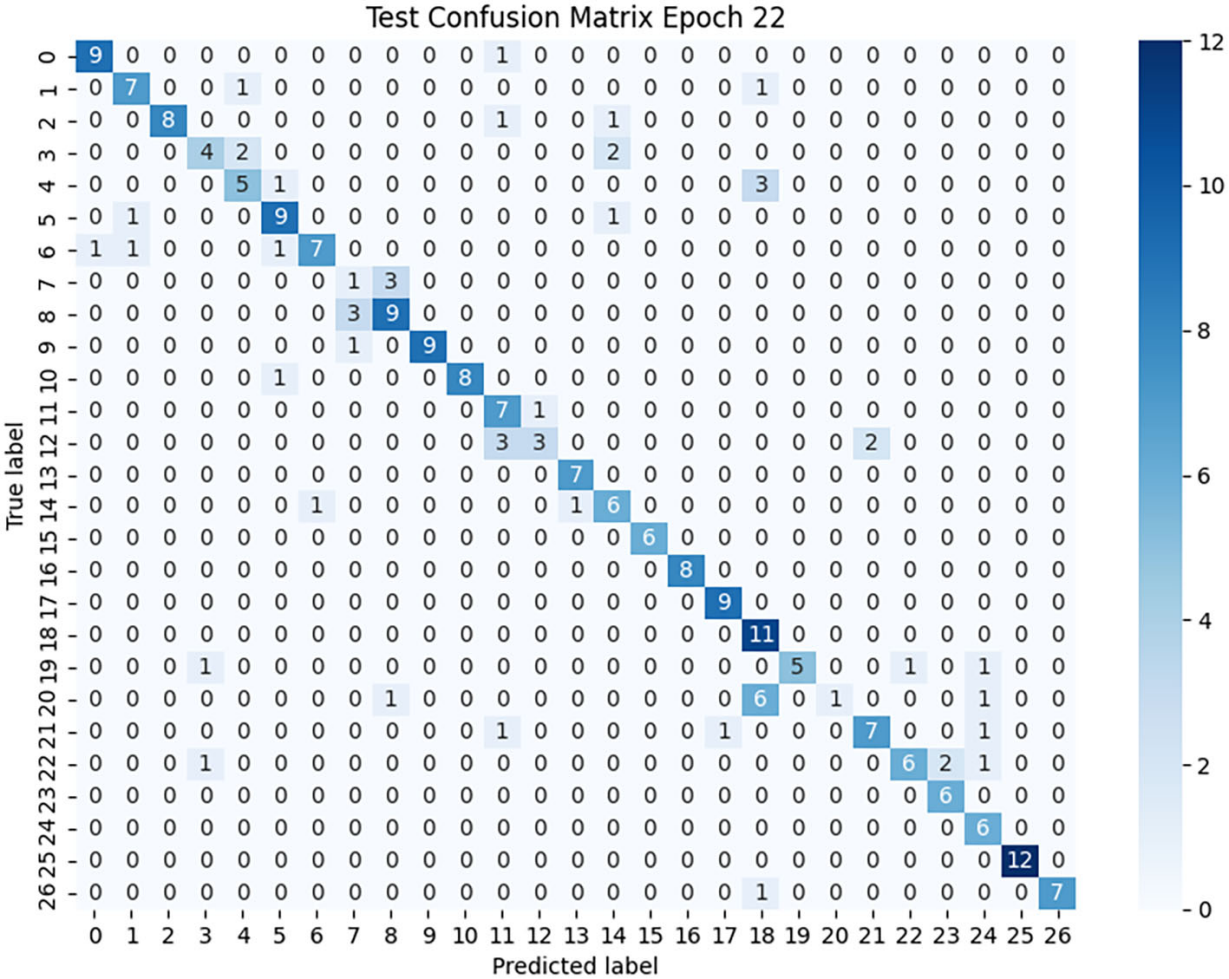
Confusion matrix heatmap for the PlantDoc test set classification.

The confusion matrix (**Figure 10**) further supports this conclusion. The concentration of values along the main diagonal shows that most predicted categories are consistent with their true labels. For instance, nearly all samples of categories 0, 16, and 25 were correctly classified, reflecting the model’s stability and robustness in feature extraction and discrimination for these categories. Meanwhile, some off-diagonal regions exhibit noticeable confusion, such as misclassifications between categories 7 and 2, confusion between 12 and 11, and the low recognition rate of category 20, revealing the model’s limitations when handling classes with low feature distinctiveness or ambiguous lesion boundaries.

**Figure 11:**
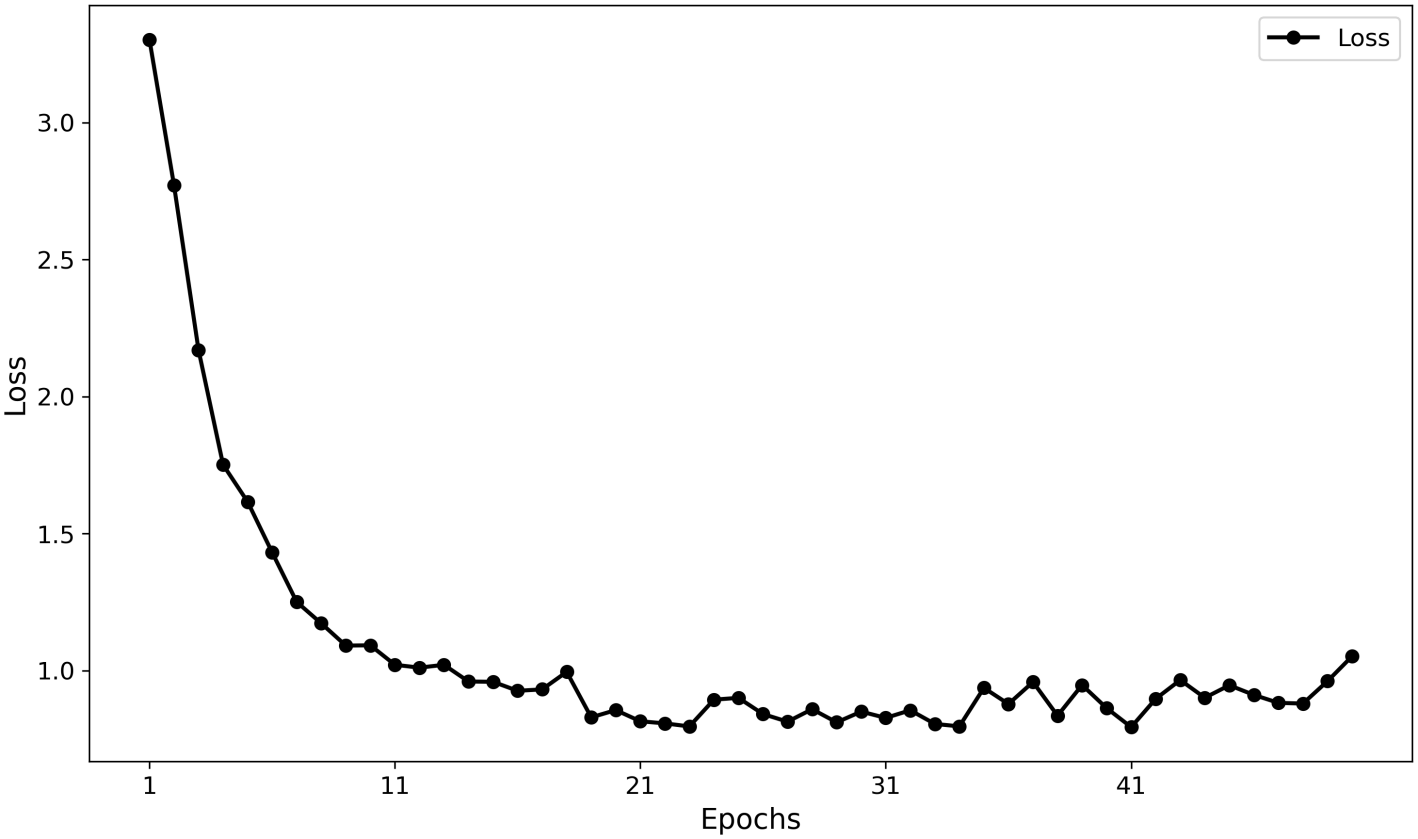
Model training loss curve over epochs.

**Figure 12:**
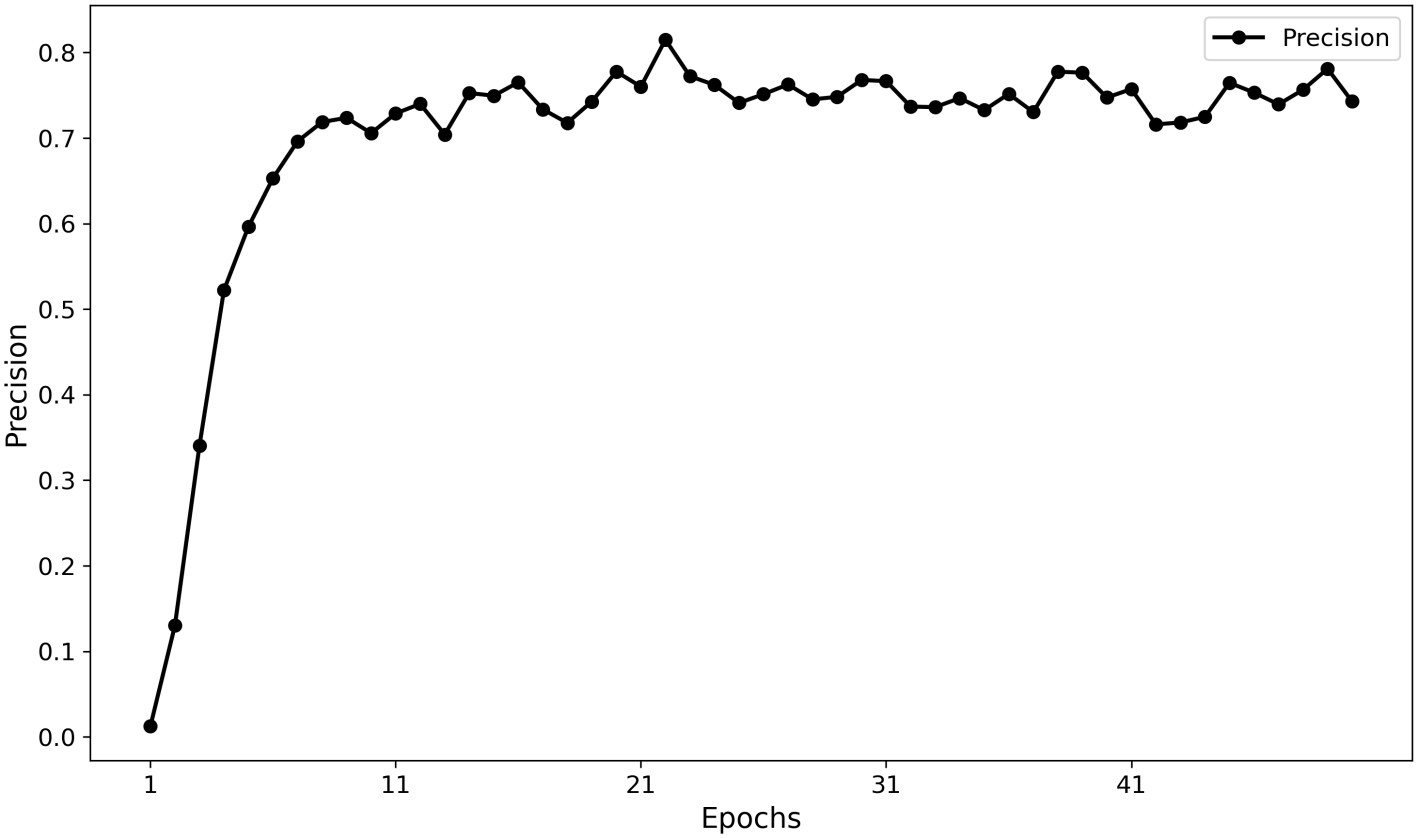
Model training precision curve over epochs.

Furthermore, the training curves (**Figures 11**, **12**) indicate that MamSwinNet possesses good convergence and feature-learning capability. During the first 10 epochs, Precision rapidly increased from 0 to over 75%, accompanied by a significant reduction in loss. After 20 epochs, the model entered a fluctuating convergence stage, with Precision stabilizing between 75% and 80% and the loss curve flattening out, suggesting that the model can efficiently learn discriminative representations and maintain stable performance within a relatively short training cycle.

Overall, MamSwinNet achieves stable recognition results across most disease categories but still shows room for improvement when dealing with complex scenes involving similar colors, blurred edges, or inconspicuous features. Future research could further enhance model performance through the following strategies: (1) introducing a multi-scale feature extraction mechanism to capture both fine-grained textures and global structural information at different scales, thereby enhancing lesion detail modeling; (2) integrating contrastive learning to enlarge the feature-space distance between lesions and background samples, improving the model’s discriminative ability for easily confused categories; (3) further optimizing the attention mechanism design to strengthen the model’s focus on key regions and reduce interference from illumination variation and background noise; and (4) exploring domain adaptation and transfer learning techniques to improve generalization across different crop types and environmental conditions.

#### 3.2.2 Comparative experiments

To comprehensively evaluate the performance of MamSwinNet in plant disease detection, we conducted detailed comparative experiments against mainstream deep learning models. The baseline models included Shufflenet (Zhang et al., 2018), EfficientNet (Atila et al., 2021), MobileNet V3 (Qian et al., 2021), MobileViT (Mehta and Rastegari, 2021), ConvNeXt (Wu et al., 2023), DenseNet (Zhu and Newsam, 2017), ResNet (Targ et al., 2016), GoogLeNet (Al-Qizwini et al., 2017), RegNetX (Guo et al., 2025), and Swin Transformer (Liu et al., 2021), along with classic Transformer architectures. In addition, models specifically designed for plant disease detection, such as T-CNN (Wang et al., 2021) and ICVT (Yu et al., 2023), were included for comparison. The results are summarized in **Table 2**.

**Table 2:** Comparison of classification performance and efficiency (parameters, Glops) for different models.

Model	Precision (%)	Recall (%)	F1 (%)	Model parameters (M)	GMACs
ShufflenetV2 ×1.0	70.15	68.29	69.21	1.2	0.15
ShufflenetV2 ×2.0	74.12	71.21	72.64	5.4	0.59
EfficientNet-B0	70.52	69.75	70.13	4.04	0.40
EfficientNett-B1	72.15	71.86	72.00	6.55	0.59
MobileNetV3-Small	58.66	56.78	57.70	1.55	0.059
MobileNetV3-Large	63.42	58.89	61.07	4.24	0.23
MobileViT-S	72.01	71.51	71.76	4.95	1.56
MobileViT-XS	73.32	69.87	71.55	1.94	0.80
MobileViT-XXS	67.56	63.14	65.27	0.86	0.24
ConvNeXt-Tiny	72.01	71.34	71.67	27.84	4.49
ConvNeXt-Small	65.96	63.89	64.91	49.48	8.73
DenseNet-121	74.10	71.63	72.84	6.98	2.90
DenseNet-161	76.72	72.88	74.75	26.53	7.85
DenseNet-201	75.08	72.32	73.67	18.14	4.39
ResNet-34	70.70	65.67	68.09	21.30	3.68
ResNet-50	74.21	67.79	70.85	23.56	4.13
GoogLeNet	70.81	65.25	67.92	10.01	1.51
regnetx-1.6	69.34	68.54	68.94	8.30	1.83
regnetx-3.2	71.24	70.56	70.89	14.31	3.62
Regnetx-4.0	74.62	74.41	74.51	20.79	4.40
Transformer Base	55.58	57.51	56.53	85.82	16.88
Swin Transformer Tiny	76.72	72.95	74.79	27.54	4.38
Swin Transformer Base	74.75	70.76	72.70	86.77	15.19
T-cnn(ResNet-101)	74.44	–	–	–	–
ICVT	77.23	–	–	–	–
MamSwinNet	81.50	77.54	79.47	12.97	2.71

In terms of core classification performance, MamSwinNet demonstrates remarkable superiority. Its Precision reaches 81.50%, Recall 77.54%, and F1-Score 79.47%, significantly outperforming most comparison models. When compared with lightweight models such as ShufflenetV2×1.0 (F1 69.21%) and MobileNetV3-Large (F1 61.07%), MamSwinNet achieves F1 improvements of 10.26 and 18.30 percentage points, respectively, overcoming the inherent precision limitations of lightweight architectures in handling complex plant disease datasets. Against classic CNN models like DenseNet-121 (F1 72.84%) and ResNet-50 (F1 70.85%), MamSwinNet leverages its ability to model long-range dependencies and cross-dimensional features, boosting F1 by 6.63 and 8.62 percentage points, respectively. For Transformer-based methods, MobileViT-XS (F1 71.55%) and Swin Transformer Base (F1 72.70%) struggle to balance accuracy and efficiency due to the high computational cost of self-attention, whereas MamSwinNet’s innovative modules improve F1 by 8.02 and 6.77 percentage points, respectively. Even plant-disease-specific models such as T-CNN (ResNet-101, Precision 74.44%) and ICVT (Precision 77.23%) fall short, with MamSwinNet surpassing them by 7.06 and 4.27 percentage points in Precision, respectively.

In terms of computational efficiency, MamSwinNet achieves an excellent balance between accuracy and resource consumption. It contains only 12.97M parameters and a computational complexity of 2.71 GMac. In contrast, DenseNet-161 (F1 74.75%) has 26.53M parameters, more than 2.04× larger, while Swin Transformer Base (F1 72.70%) requires 15.19 GMac, which is approximately 5.6× higher than MamSwinNet.

Taken together, MamSwinNet comprehensively outperforms mainstream models in both accuracy and efficiency, highlighting its distinctive advantages and broad applicability for plant disease detection tasks.

#### 3.2.3 Ablation study: module contribution analysis

To quantify the independent contributions and synergistic effects of each core module in MamSwinNet, four groups of ablation experiments were conducted (**Table 3**). Using Swin-T as the baseline, the comparison was carried out in terms of both classification performance (Precision, Recall, F1-Score) and computational efficiency (number of parameters and GMACs).

**Table 3:** Ablation experiment results.

Experiment	Precision (%)	Recall (%)	F1 (%)	Model parameters	GMACs
1	76.78	74.15	75.44	27.54	4.38
2	78.54	75.91	77.20	16.68	3.09
3	79.74	76.27	77.97	12.93	2.71
4	78.38	75.33	76.82	12.94	2.70
5	80.01	76.75	78.35	14.85	2.93
6	81.50	77.54	79.47	12.97	2.71

Experiment 1: Baseline Model (Swin-T).

Using Swin Transformer Tiny (Swin-T) as the benchmark, the model achieved a Precision of 76.78%, Recall of 74.15%, and F1-Score of 75.44%. Although it demonstrated basic classification capability, its large parameter count (27.54M) and computational cost (4.38 GMac) impose significant limitations on efficient deployment in real-world agricultural scenarios, highlighting the necessity of further optimization.

Experiment 2: Removal of the Efficient Token Refinement Module.

When the SGSP and CCGOS modules were retained but Efficient Token Refinement was removed, model accuracy improved slightly (Precision 78.54%, Recall 75.91%, F1-Score 77.20%). However, parameters increased to 16.68M and complexity to 3.09 GMac, indicating that this module plays a crucial role in reducing parameters and computational overhead. Its removal yields marginal accuracy gains but undermines the lightweight advantage.

Experiment 3: Removal of the SGSP Module.

Retaining Efficient Token Refinement and CCGOS while removing SGSP resulted in Precision 79.74%, Recall 76.27%, and F1-Score 77.97%. Parameter count dropped to 12.93M and computational cost to 2.71 GMac, closely matching the efficiency of the complete model. However, the F1-Score decreased by 1.50 percentage points, underscoring the indispensable role of SGSP in modeling long-range spatial dependencies, which is key to improving classification accuracy for complex plant diseases.

Experiment 4: Removal of the CCGOS Module.

When Efficient Token Refinement and SGSP were retained but CCGOS removed, Precision declined to 78.38%, Recall to 75.33%, and F1-Score to 76.82%. Parameters totaled 12.94M with 2.70 GMac, but this setting produced the lowest accuracy among all “single-module removal” experiments. This confirms that CCGOS is irreplaceable for channel–spatial feature fusion.

Experiment 5: Replacing SGSP and CCGOS with Standard Transformer Blocks.

Replacing SGSP and CCGOS with standard Transformer modules while retaining Efficient Token Refinement produced Precision 80.01%, Recall 76.75%, and F1-Score 78.35%. However, parameters increased to 14.85M and complexity to 2.93 GMac. Although accuracy exceeded the baseline, it was 1.12 percentage points lower than the complete model, with greater computational cost. This suggests that while standard Transformer blocks provide basic feature modeling, they lack the efficiency and task-specific adaptability of SGSP and CCGOS.

Experiment 6: Complete MamSwinNet.

The full integration of Efficient Token Refinement, SGSP, and CCGOS yielded the best performance: Precision 81.50%, Recall 77.54%, and F1-Score 79.47%, with only 12.97M parameters and 2.71 GMac. Compared to the baseline, parameters were reduced by 52.8% and computation by 38.1%, alongside significant accuracy gains. These results highlight the synergistic effect of the three modules: Efficient Token Refinement optimizes token quality and reduces redundancy, SGSP enhances long-range spatial dependency modeling, and CCGOS strengthens channel–spatial feature fusion. Collectively, they achieve a dual breakthrough in accuracy enhancement and efficiency optimization, demonstrating the necessity and superiority of the complete architecture.

#### 3.2.4 Model visualization analysis based on Grad-CAM

To further assess MamSwinNet’s capacity for lesion-focused feature capture and decision interpretability, this section employs Gradient-weighted Class Activation Mapping (Grad-CAM) on representative disease samples to visualize attention heatmaps during classification. In these maps, colors from blue to red indicate increasing feature contribution. A greater overlap between red regions and the ground-truth lesions signifies more accurate focus on disease-critical features during decision-making, thereby providing intuitive, visual evidence of the model’s reasoning process.

**Figure 13:**
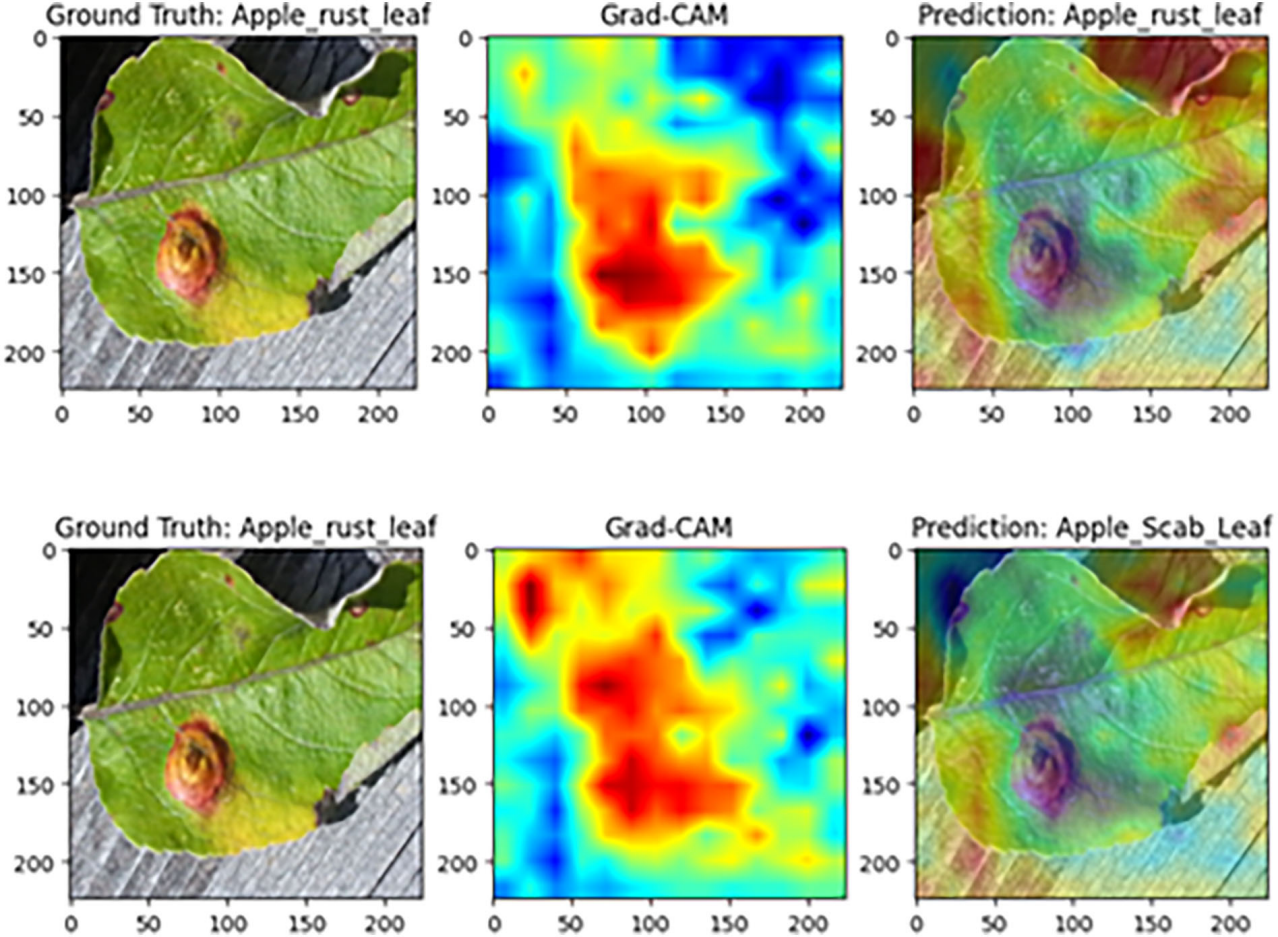
Grad-CAM comparison on apple rust disease samples (Top: complete MamSwinNet model; Bottom: after removing the Efficient Token Refinement module).

**Figure 13** presents a Grad-CAM comparison on apple rust samples. In the complete model, high-contribution (red) regions align closely with the reddish-brown, near-circular raised lesions on the leaf surface in both location and morphology, leading to a correct classification as “apple rust leaf.” However, after removing the Efficient Token Refinement module, the coverage of red regions over the lesions declines markedly, lesion boundaries become blurred, and the model ultimately misclassifies the sample as “apple black spot leaf.” This comparison indicates that the Efficient Token Refinement module plays a pivotal role in preserving the spatial structural information of lesion boundaries, enabling precise focus on disease-core regions and supporting correct classification.

**Figure 14:**
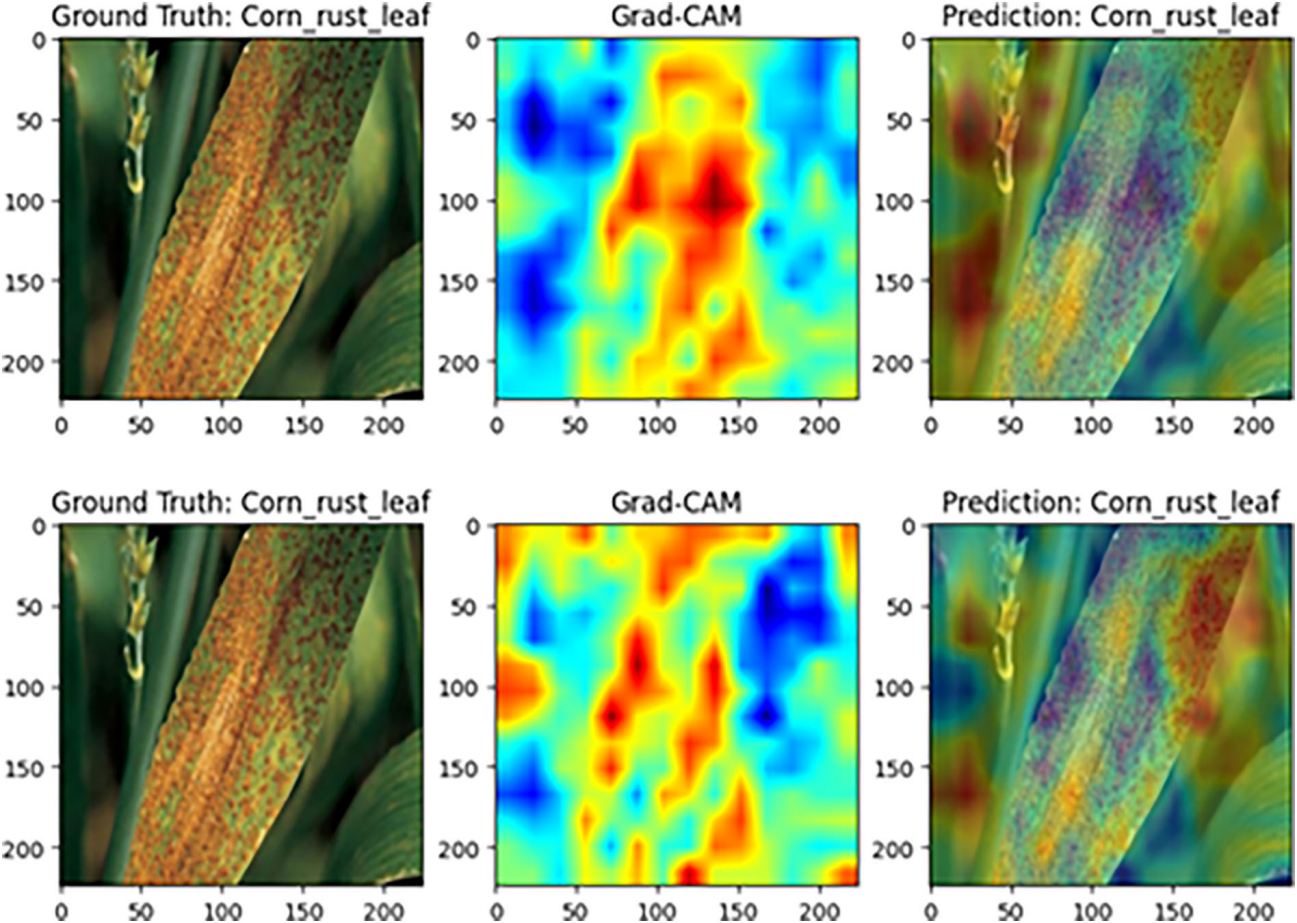
Grad-CAM comparison on maize rust disease samples (Top: complete MamSwinNet model; Bottom: after removing the SGSP module).

**Figure 14** shows the visualization for maize rust samples. With the complete model, high-contribution regions are highly consistent with the diffusely distributed rust pustules across the leaf, yielding an accurate classification as “maize rust leaf.” After the SGSP module is ablated, the heatmap becomes fragmented and lacks continuity. Although the final label remains “maize rust leaf,” the model’s overall coverage and focus on the dispersed lesion pattern are substantially reduced. This suggests that SGSP is crucial for modeling long-range dependencies characteristic of diffuse lesion distributions.

**Figure 15:**
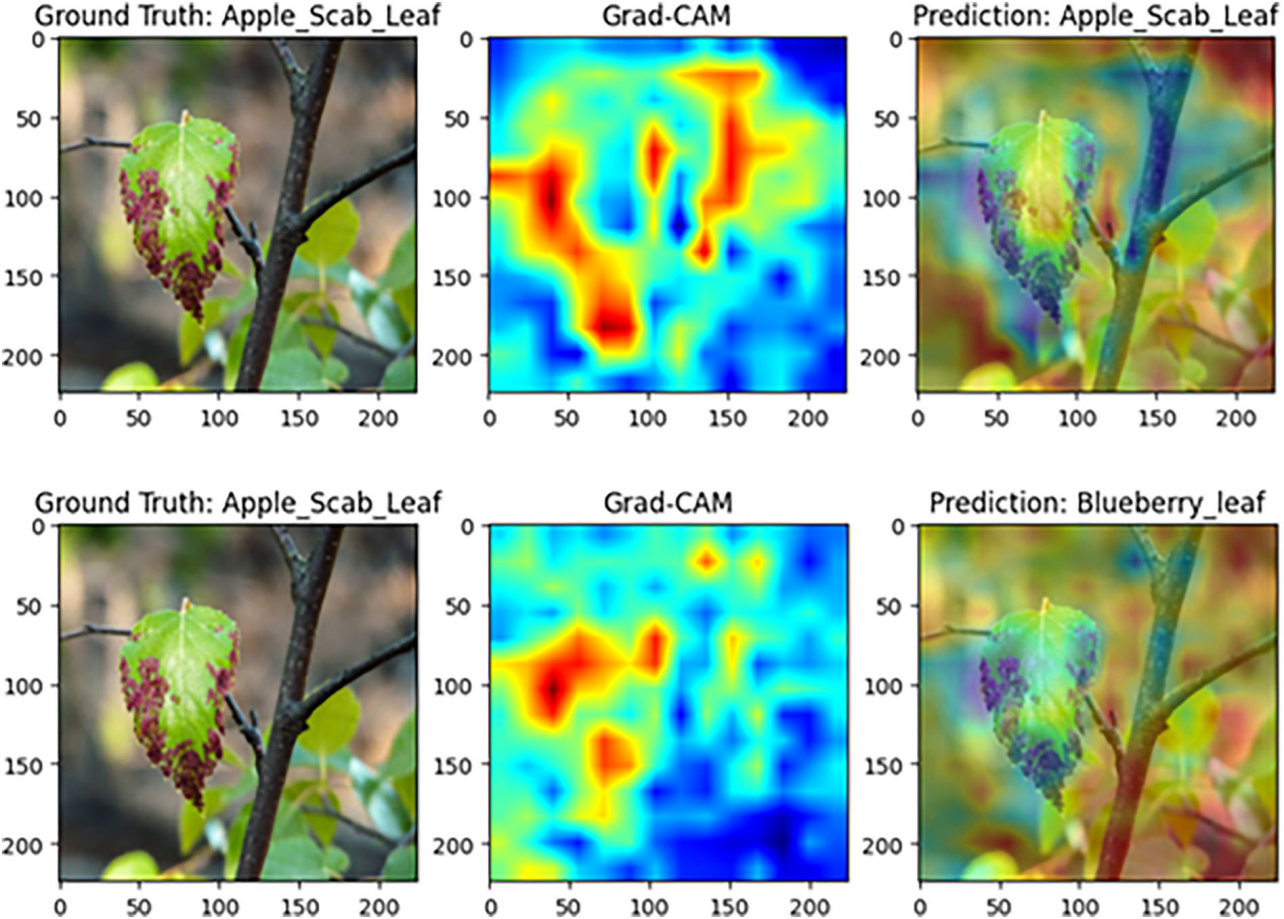
Grad-CAM comparison on apple black spot disease samples (Top: complete MamSwinNet model; Bottom: after removing the CCGOS module).

**Figure 15** illustrates the results for apple black spot samples. Under the complete model, the high-contribution regions align closely with black spot lesions along the leaf margin, resulting in a correct prediction of “apple black spot leaf.” In contrast, removing the CCGOS module significantly diminishes focus on the lesion area, shifts the attention distribution, and leads to a misclassification as “blueberry leaf.” These findings indicate that CCGOS is essential for joint channel–spatial feature modeling, effectively suppressing background interference and highlighting discriminative lesion cues. Without this module, the model becomes more prone to confusion in complex backgrounds, degrading classification accuracy.

In summary, the interpretability analysis of MamSwinNet demonstrates that its attention distribution aligns closely with the actual lesion regions. The three key modules play essential roles in preserving spatial structure, modeling long-range dependencies, and enhancing channel–spatial feature fusion, thereby improving both the accuracy and robustness of plant disease recognition. These findings are consistent with the high performance reported in the experimental results, further underscoring the robustness and practical value of MamSwinNet.

#### 3.2.5 Impact of regularization methods on model performance

To explore the regulatory effect of regularization strategies on the feature learning preferences and generalization ability of the MamSwinNet model, comparative experiments were designed using three configurations: L1 regularization, L2 regularization, and no regularization. The evaluation was conducted on the PlantDoc dataset using Precision, Recall, and F1-Score (**Table 4**).

**Table 4:** Comparison of classification performance indicators for L1, L2, and no regularization strategies.

Regularization methods	Precision (%)	Recall (%)	F1 (%)
L1	81.50	77.54	79.47
L2	80.51	76.49	78.45
No Regularization	79.97	75.86	77.86

The experimental results show that L1 regularization significantly improved the model’s performance: Precision reached 81.50%, Recall was 77.54%, and F1-Score was 79.47%, outperforming both L2 regularization (Precision 80.51%, Recall 76.49%, F1-Score 78.45%) and no regularization (Precision 79.97%, Recall 75.86%, F1-Score 77.86%). The core mechanism behind this lies in the sparsity-inducing nature of L1 regularization: by applying L1 norm penalties to the weights, the model tends to learn highly discriminative “core features” (such as the edge contours of lesions and the specific response of color channels) while suppressing redundant noise features (such as the vein texture of the background leaf or lighting interference), allowing the feature representation to focus more on the essential patterns of the disease.

In contrast, L2 regularization uses a global weight decay strategy, which can alleviate overfitting by reducing the weight scale, but due to the lack of feature selection, it may excessively compress fine-grained features of the disease spot (such as the texture of tiny spots), leading to a decrease in Recall (1.05 percentage points lower than L1). In the case of no regularization, the model tends to overfit sample-level noise in the training set (such as leaf wrinkles and shadows in the images), which results in poor generalization ability on the test set (F1-Score is 1.61 percentage points lower than L1), further confirming the critical role of regularization in preventing overfitting.

In summary, L1 regularization, through “sparse feature selection,” adapts to the heterogeneity of plant disease features (such as lesion shapes and color diversity), enhancing model robustness while improving classification accuracy, making it an efficient strategy for optimizing the MamSwinNet model’s performance in disease detection.

#### 3.2.6 Token dimensions and quantity impact on model performance

To explore the impact of token dimensions and quantity in the Efficient Token Refinement module on model classification performance and computational efficiency, experiments were conducted by adjusting token quantities (64, 256, 324, 400) and dimensions (32, 64, 128, 256). The system analyzed Precision, Recall, F1-Score, and computational cost (model parameters, Glops) for different configurations, with results shown in **Table 5**.

**Table 5:** Model performance and efficiency (Parameters, Glops) for different token quantity and dimension combinations.

Token num	Token dim	Precision (%)	Recall (%)	F1 (%)	Model parameters	GMACs
64	32	76.14	73.45	74.77	12.30	2.62
64	64	77.09	74.88	75.97	12.48	2.65
64	128	81.50	77.54	79.47	12.97	2.71
256	128	75.34	73.45	74.38	12.98	2.72
324	256	79.20	75.42	77.26	13.03	2.79
400	256	78.62	77.11	77.86	13.05	2.81

At the same token quantity, increasing the dimension significantly improved performance. For example, with a fixed token quantity of 64, when the dimension increased from 32 to 128, the model’s performance gradually optimized:At dimension 32, the F1-Score was 74.77%, with 12.30M parameters and 2.62 Glops. At dimension 64, the F1-Score increased to 75.97%, with slight increases in parameters and Glops. At dimension 128, the F1-Score reached 79.47%, with Precision and Recall at 81.50% and 77.54%, respectively, 12.97M parameters, and 2.71 Glops, showing significant performance improvement and controllable computational cost compared to dimension 32.

This indicates that appropriately increasing the token dimension enhances feature expression, better capturing complex features such as lesion color gradients and texture details. Particularly at dimension 128, it achieved a good balance between precision and efficiency. At the same dimension, increasing the token quantity did not lead to continuous performance improvement. For instance, with a fixed dimension of 128:When the token quantity was 64, the F1-Score was 79.47%.Increasing the token quantity to 256 caused the F1-Score to drop to 74.38%, with parameters and Glops remaining relatively stable. Increasing to 324 and 400 led to a slight recovery in F1-Score (77.26% and 77.86%), but still lower than the performance with 64 tokens, while Glops increased to 2.79 and 2.81, adding to the computational burden. This suggests that too many tokens introduce redundant information (such as background noise), reducing the model’s focus on key disease features and increasing unnecessary computations.

Overall, the configuration with 64 tokens and 128 dimensions performed optimally: F1-Score of 79.47%, 12.97M parameters, and 2.71 Glops, effectively balancing high classification accuracy with manageable computational cost, making it suitable for large-scale deployment in agricultural scenarios. This result reveals that the token configuration must balance feature expression capabilities with computational efficiency, where increasing the dimension moderately improves performance, but too many tokens are counterproductive.

#### 3.2.7 Generalization experiments

To systematically evaluate the adaptability of MamSwinNet under heterogeneous data distributions and across different disease types, two representative datasets were selected for generalization experiments and compared against multiple mainstream models (results in **Tables 6**, **7**). The PlantVillage dataset, covering 14 crops and 38 diseases with over 50,000 samples, was used to test the model’s ability to capture shared disease features across multiple crops. In contrast, the Cotton Disease dataset focuses on crop-specific lesion morphologies, providing a more rigorous evaluation of adaptability in specialized scenarios.

**Table 6:** Generalization experiment results on the PlantVillage dataset.

Model	Precision (%)	Recall (%)	F1 (%)
PlantViT (Thakur et al., 2021)	98.24	98.33	98.28
ConvViT-S (Utku et al., 2025)	97.80	98.54	98.17
TLMVIT	98.72	98.76	98.73
pre-trained MobileNet-V2 and attention mechanism (Chen et al., 2021)	97.49	95.83	96.65
MamSwinNet	99.53	99.52	99.52

**Table 7:** Generalization experiment results on the cotton disease dataset.

Model	Precision (%)	Recall (%)	F1-Score (%)	Model parameters	GMACs
Swin transformer Base	98.88	98.74	98.81	86.78	15.19
Swin transformer Tiny	98.54	98.53	98.53	27.55	4.38
Transformer Base	95.68	95.50	95.59	85.83	16.88
MamSwinNet	99.32	99.44	99.38	12.97	2.71

MamSwinNet achieved outstanding performance with Precision 99.53%, Recall 99.52%, and F1-Score 99.52%, outperforming all comparison models. Compared with PlantViT (98.28%), ConvViT-S (98.17%), and TLMViT (98.73%), it achieved improvements of 1.25, 1.35, and 0.79 percentage points, respectively. Its advantage was even more pronounced over the hybrid MobileNet-V2 + attention mechanism (96.65%). These results demonstrate that MamSwinNet can effectively aggregate generalized lesion features across diverse crops, enabling accurate cross-crop classification.

MamSwinNet’s advantages were even more striking on the Cotton Disease dataset, where it achieved Precision 99.32%, Recall 99.44%, and F1-Score 99.38%, ranking first among all models. Compared with Swin Transformer Base (F1-Score 98.81%, 86.78M parameters, 15.19 GMacs), MamSwinNet not only improved the overall F1 by 0.57 percentage points, but also increased Precision and Recall by 0.44% and 0.70%, respectively. Relative to Swin Transformer Tiny (F1-Score 98.53%, 27.55M parameters, 4.38 GMacs) and Transformer Base (F1-Score 95.59%, 85.83M parameters, 16.88 GMacs), its superiority was even more evident. Notably, MamSwinNet achieved these results with only 12.97M parameters and 2.71 GMacs, significantly lower than its competitors.

These findings confirm that MamSwinNet achieves state-of-the-art performance while maintaining lightweight design and computational efficiency. Its ability to generalize across both broad multi-crop datasets and highly specialized disease scenarios highlights its strong robustness, adaptability, and suitability for real-world agricultural applications.

## 4 Conclusion

This study proposes MamSwinNet, a novel model tailored for plant disease detection, which addresses key challenges by integrating the hierarchical architecture of Swin Transformer with three innovative modules: Efficient Token Refinement, SGSP, and CCGOS. The model establishes a four-stage feature extraction framework that balances detection accuracy and computational efficiency. In the first three stages, patch partitioning, shifted window attention, and patch merging progressively construct multi-scale local representations, laying the foundation for high-level semantic learning. The fourth stage incorporates the proposed modules to specifically tackle long-range dependency modeling and cross-dimensional feature fusion: Efficient Token Refinement improves token quality and efficiency, SGSP enhances spatial global-context perception, and CCGOS enables precise channel–spatial feature integration.

Experimental validation demonstrates the superiority of MamSwinNet. On the PlantDoc dataset, the model achieves Precision 81.50%, Recall 77.54%, and F1-Score 79.47%, outperforming mainstream models such as Shufflenet, MobileNet, and Swin Transformer. Its cross-dataset generalization ability is also remarkable, maintaining high accuracy on both PlantVillage (F1 99.52%) and Cotton Disease (F1 99.38%) datasets. Ablation experiments confirm the complementary synergy of the proposed modules: compared to the Swin-T baseline, the complete model reduces parameters by 52.8% and computation by 38.1%, while boosting the F1-Score by 4.03 percentage points. Further, token configuration and regularization studies identify 128-dimensional tokens with a quantity of 64 and L1 regularization as the optimal setting, underscoring the model’s practical deployment value.

Future research can focus on several key areas to further enhance the performance of the MamSwinNet model. First, optimizing the model’s real-time response capability is crucial, especially for large-scale agricultural disease monitoring systems. By improving the inference process and accelerating model computation, the system’s processing speed can be increased to meet real-time monitoring demands. Second, as environments and crop types diversify, future studies should focus on improving the model’s adaptability to complex environments. Techniques such as transfer learning and domain adaptation can be employed to enhance the model’s generalization capability across different crops and pest types, ensuring its efficient application in real-field scenarios. Moreover, combining other advanced deep learning technologies, such as reinforcement learning and Generative Adversarial Networks (GANs), can further increase the model’s flexibility and robustness, enabling it to handle more complex disease detection tasks. These research directions will lay the foundation for the further optimization and widespread application of the MamSwinNet model.

## Data Availability

Publicly available datasets were analyzed in this study. This data can be found here: Cotton Plant Disease Dataset Repository: Kaggle Direct link: https://www.kaggle.com/datasets/dhamur/cotton-plant-disease PlantDoc Dataset Repository: GitHub Direct link: https://github.com/Sana-Gupta/PlantDoc-Dataset PlantVillage Dataset Repository: Kaggle Direct link: https://www.kaggle.com/datasets/abdallahalidev/plantvillage-dataset.

## References

[B1] Ali M. Salma M. Haji M. E. Jamal B. (2025). Plant disease detection using vision transformers. Int. J. Electrical Comput. Eng. (IJECE) 15, 2334–2344. doi: 10.11591/ijece.v15i2.pp2334-2344

[B2] Al-Qizwini M. Barjasteh I. Al-Qassab H. Radha H. (2017). “Deep learning algorithm for autonomous driving using GoogLeNet,” in Proceedings of the 2017 IEEE Intelligent Vehicles Symposium (IV). 89–96 (IEEE).

[B3] Ashurov A. Y. Al-Gaashani M. S. A. M. Samee N. A. Alkanhel R. Atteia G. Abdullah H. A.. (2025). Enhancing plant disease detection through deep learning: a Depthwise CNN with squeeze and excitation integration and residual skip connections. Front. Plant Sci. 15, 1505857. doi: 10.3389/fpls.2024.1505857, PMID: 39925367 PMC11803862

[B4] Atila Ü. Uçar M. Akyol K. Uçar E. (2021). Plant leaf disease classification using EfficientNet deep learning model. Ecol. Inf. 61, 101182. doi: 10.1016/j.ecoinf.2020.101182

[B5] Baek E. T. (2025). Attention score-based multi-vision transformer technique for plant disease classification. Sensors 25, 270. doi: 10.3390/s25010270, PMID: 39797061 PMC11723448

[B6] Bhargava R. Upadhyay S. K. Sharma R. Srivastava N. Mavi H. (2024). “Plant Disease detection using Machine learning and CNN on leaf images,” in Proceedings of the 2024 1st International Conference on Advanced Computing and Emerging Technologies (ACET). 1–7 (IEEE).

[B7] Chen J. Zhang D. Zeb A. Nanehkaran Y. A. (2021). Identification of rice plant diseases using lightweight attention networks. Expert Syst. Appl. 169, 114514. doi: 10.1016/j.eswa.2020.114514

[B8] Duhan S. Gulia P. Gill N. S. Yahya M. Yadav S. Hassan M. M.. (2024). An analysis to investigate plant disease identification based on machine learning techniques. Expert Syst. 41, e13576. doi: 10.1111/exsy.13576

[B9] Guo L. Huang S. Xin B. (2025). MS-RegNet: A rock image classification algorithm based on improved RegNet. Leading Edge 44, 33–42. doi: 10.1190/tle44010033.1

[B10] Kusumo B. S. Heryana A. Mahendra O. Pardede H. F. (2018). “Machine learning-based for automatic detection of corn-plant diseases using image processing,” in Proceedings of the 2018 International Conference on Computer, Control, Informatics and Its Applications (IC3INA). 93–97 (IEEE).

[B11] Liu Z. Lin Y. Cao Y. Hu H. Wei Y. Zhang Z.. (2021). “Swin transformer: Hierarchical vision transformer using shifted windows,” in Proceedings of the IEEE/CVF International Conference on Computer Vision. 10012–10022.

[B12] Mehta S. Rastegari M. (2021). MobileViT: Light-weight, general-purpose, and mobile-friendly vision transformer. arXiv Preprint arXiv:2110.02178. doi: 10.48550/arXiv.2110.02178

[B13] Ouamane A. Chouchane A. Himeur Y. Debilou A. Nadji S. Boubakeur N.. (2024). Enhancing plant disease detection: A novel CNN-based approach with tensor subspace learning and HOWSVD-MDA. Neural Computing Appl. 36, 22957–22981. doi: 10.1007/s00521-024-10454-1

[B14] Panchal P. Raman V. C. Mantri S. (2019). “Plant diseases detection and classification using machine learning models,” in Proceedings of the 2019 4th International Conference on Computational Systems and Information Technology for Sustainable Solution (CSITSS). 1–6 (IEEE).

[B15] Phadikar S. (2012). Classification of rice leaf diseases based on morphological changes. Int. J. Geographical Inf. Sci. 2. doi: 10.7763/ijiee.2012.v2.137

[B16] Qian S. Ning C. Hu Y. (2021). “MobileNetV3 for image classification,” in Proceedings of the 2021 IEEE 2nd International Conference on Big Data, Artificial Intelligence and Internet of Things Engineering (ICBAIE). 490–497 (IEEE).

[B17] Rakib A. F. Rahman R. Razi A. A. Hasan A. S. M. T. (2024). A lightweight quantized CNN model for plant disease recognition. Arabian J. Sci. Eng. 49, 4097–4108. doi: 10.1007/s13369-023-08280-z

[B18] Targ S. Almeida D. Lyman K. (2016). ResNet in ResNet: Generalizing residual architectures. arXiv Preprint arXiv:1603.08029. doi: 10.48550/arXiv.1603.08029

[B19] Thakur P. S. Khanna P. Sheorey T. Ojha A. (2021). “Vision transformer for plant disease detection: PlantViT,” in Proceedings of the International Conference on Computer Vision and Image Processing (Berlin and Heidelberg, Germany: Springer International Publishing), 501–511.

[B20] Utku A. Kaya M. Canbay Y. (2025). A new hybrid ConvViT model for dangerous farm insect detection. Appl. Sci. 15, 2518. doi: 10.3390/app15052518

[B21] Wang D. Wang J. Li W. Guan P. (2021). T-CNN: Trilinear convolutional neural networks model for visual detection of plant diseases. Comput. Electron. Agric. 190, 106468. doi: 10.1016/j.compag.2021.106468

[B22] Wu Q. Ma X. Liu H. Bi C. Yu H. Liang M.. (2023). A classification method for soybean leaf diseases based on an improved ConvNeXt model. Sci. Rep. 13, 19141. doi: 10.1038/s41598-023-46492-3, PMID: 37932395 PMC10628197

[B23] Yu S. Xie L. Huang Q. (2023). Inception convolutional vision transformers for plant disease identification. Internet Things 21, 100650. doi: 10.1016/j.iot.2022.100650

[B24] Zhang Z. Gong Z. Hong Q. Jiang L. (2021). “Swin-transformer based classification for rice diseases recognition,” in Proceedings of the 2021 International Conference on Computer Information Science and Artificial Intelligence (CISAI). 153–156 (IEEE).

[B25] Zhang X. Zhou X. Lin M. Sun J. (2018). “Shufflenet: An extremely efficient convolutional neural network for mobile devices,” in Proceedings of the IEEE Conference on Computer Vision and Pattern Recognition. 6848–6856.

[B26] Zhu Y. Newsam S. (2017). “DenseNet for dense flow,” in Proceedings of the 2017 IEEE International Conference on Image Processing (ICIP). 790–794 (IEEE).

